# No more helper adenovirus: production of gutless adenovirus (GLAd) free of adenovirus and replication-competent adenovirus (RCA) contaminants

**DOI:** 10.1038/s12276-019-0334-z

**Published:** 2019-10-28

**Authors:** Dongwoo Lee, Jida Liu, Hyun Jung Junn, Eun-Joo Lee, Kyu-Shik Jeong, Dai-Wu Seol

**Affiliations:** 1 Genenmed Inc., 84 Seongsuil-ro, Seongdong-gu, Seoul, Republic of Korea; 2 College of Pharmacy, Chung-Ang University, Seoul, Republic of Korea; 3 Department of Veterinary Pathology, College of Veterinary Medicine, Kyungpook National University, Daegu City, Republic of Korea

**Keywords:** Gene therapy, Gene delivery

## Abstract

Gene therapy is emerging as an effective treatment option for various inherited genetic diseases. Gutless adenovirus (GLAd), also known as helper-dependent adenovirus (HDAd), has many notable characteristics as a gene delivery vector for this particular type of gene therapy, including broad tropism, high infectivity, a large transgene cargo capacity, and an absence of integration into the host genome. Additionally, GLAd ensures long-term transgene expression in host organisms owing to its minimal immunogenicity, since it was constructed following the deletion of all the genes from an adenovirus. However, the clinical use of GLAd for the treatment of inherited genetic diseases has been hampered by unavoidable contamination of the highly immunogenic adenovirus used as a helper for GLAd production. Here, we report the production of GLAd in the absence of a helper adenovirus, which was achieved with a helper plasmid instead. Utilizing this helper plasmid, we successfully produced large quantities of recombinant GLAd. Importantly, our helper plasmid-based system exclusively produced recombinant GLAd with no generation of helper plasmid-originating adenovirus and replication-competent adenovirus (RCA). The recombinant GLAd that was produced efficiently delivered transgenes regardless of their size and exhibited therapeutic potential for Huntington’s disease (HD) and Duchenne muscular dystrophy (DMD). Our data indicate that our helper plasmid-based GLAd production system could become a new platform for GLAd-based gene therapy.

## Gene therapy: Production of next-generation ‘gutless’ adenovirus vectors

A new protocol allows for the manufacturing of a next-generation gene therapy vector without contamination of helper adenovirus and replication-competent adenovirus (RCA). Adenoviruses are often used to deliver therapeutic DNA, but their proteins can trigger immune reactions. So-called ‘gutless’ adenoviruses that lack all viral genes don’t cause the same problem but their production has traditionally relied on a helper adenovirus that remains as an unavoidable contaminant. A team led by Dai-Wu Seol from Chung-Ang University in Seoul, South Korea, has now prepared large quantities of gutless adenoviruses using helper plasmid, a circular DNA that encodes all the proteins needed for production of gutless adenoviruses but do not leave behind any contaminant adenoviruses. Gutless adenoviruse vectors made this way successfully delivered corrected copies of the faulty genes responsible for human diseseas into human cells and mice.

## Introduction

Gene therapy that recovers the normal function of the target gene by replacing the corresponding defective gene is emerging as an effective therapeutic option for various inherited genetic diseases, including Leber’s congenital amaurosis (LCA)^1,2^ and spinal muscular atrophy (SMA)^3^. This particular type of gene therapy depends upon a vehicle referred to as a vector for delivery of the functional gene to the target tissues or organs.

In gene therapy for the treatment of inherited genetic diseases, two types of gene delivery can be considered: ex vivo and in vivo. Ex vivo gene delivery is an approach for cell therapy that utilizes genetically engineered cells. In this approach, vector safety can be continuously monitored at different stages prior to the implantation of genetically manipulated cells into the patient’s body. In contrast, in vivo gene delivery directly transfers a therapeutic transgene to patient tissues or organs as the final destined location; thus, vector safety is more critical for this method. In addition to being safe, in vivo gene delivery vectors should ensure long-term transgene expression to sustain the therapeutic efficacy of the delivered transgene for a long period of time.

Currently, the most commonly used in vivo gene delivery vector for the clinical treatment of inherited genetic diseases is adeno-associated virus (AAV)^4–6^. The safety of AAV has been well established through a wide variety of clinical trials. AAV exhibits broad tropism for infection and ensures long-term transgene expression in various tissues and organs. These characteristics have drawn considerable attention toward AAV as an in vivo gene delivery vector for various inherited genetic diseases. Nevertheless, AAV has two notable drawbacks: potential for insertional mutagenesis and a low packaging capacity. AAV randomly integrates into the host genome^7^, although it mainly remains as an episome, and its integration frequency is low. This characteristic raises concern about insertional mutagenesis. AAV also possesses a small transgene cargo capacity (~4.5 kb)^8,9^ and cannot deliver large genes such as *huntingtin* (9.4 kb) or *dystrophin* (11 kb) or multiple genes. These aspects suggest that an in vivo gene delivery vector with a high safety profile and large transgene cargo capacity but no ability to randomly integrate into the host genome is more desirable, and such a vector could provide better opportunities for in vivo gene therapy.

Gutless adenovirus (GLAd), also known as helper-dependent adenovirus (HDAd), has been considered as a last-generation adenovirus^10–13^. GLAd is constructed following the deletion of all the genes from an adenovirus, resulting in no expression of adenoviral proteins. This structural characteristic minimizes the host immune response and allows long-term transgene expression in host tissues or organs^14–19^. GLAd also shows broad tropism for infection and a high transduction efficiency in transgene delivery. In fact, GLAd is highly comparable to AAV in terms of many safety issues. Moreover, GLAd presents prominent advantages over AAV in regard to genome integration and transgene cargo capacity^10–13,20^. GLAd does not integrate into the host genome, which eliminates concern about insertional mutagenesis. GLAd also exhibits a high accommodation capacity (up to 36 kb) for transgenes, hence making it possible to deliver large genes and multiple genes.

However, despite its many evident beneficial features, there is a problem associated with the production of the currently available GLAd. Since GLAd is devoid of all adenoviral genes, the production of recombinant GLAd is absolutely dependent upon a helper adenovirus^21–24^ that provides all viral proteins for GLAd packaging. In the standard production process, the helper adenovirus actively replicates while providing helper function and remains as a contaminant in the final GLAd preparation. Although a significant reduction of contaminant helper adenovirus can be achieved through Cre-loxP-based excision of the Ψ packaging signal, complete removal of contaminant helper adenovirus in GLAd production is very difficult to achieve^21–24^. Moreover, the helper adenovirus can generate a replication-competent adenovirus (RCA) through homologous recombination between helper adenovirus and the E1 region present in packaging cells^21^. These undesirable contaminant helper adenovirus and RCA can cause serious acute and chronic toxicity in host organisms. Furthermore, the host immune response against viral proteins expressed from these contaminant viruses can kill the cells co-infected with recombinant GLAd and these contaminant viruses, which could eventually cause the expression of GLAd-mediated therapeutic transgenes to deteriorate. These unavoidable problems have raised safety concerns and hindered the clinical use of GLAd despite its unique features and tremendous advantages. Therefore, it is vital to establish a system that can produce recombinant GLAd in the absence of helper adenovirus, resulting in no contamination of helper adenovirus and RCA.

Here, we report the production of GLAd in the absence of helper adenovirus. The helper function for GLAd packaging and further amplification is provided by a helper plasmid that does not contain any *cis*-acting elements required for virus packaging. In accordance with its structural characteristics, this helper plasmid cannot be converted into an adenovirus. Utilizing this helper plasmid, we successfully produced large quantities of recombinant GLAd that was free of adenovirus and RCA contaminants. The recombinant GLAd that was produced efficiently delivered many target transgenes and exhibited therapeutic potential for Huntington’s disease (HD) and Duchenne muscular dystrophy (DMD). Our new GLAd production system could provide opportunities for the clinical application of GLAd-based gene therapy for various inherited genetic diseases.

## Materials and methods

### Reagents, kits, mice, and general cloning techniques

All the restriction enzymes, Klenow fragment and *Hin*dIII-digested lambda phage DNA were purchased from New England Biolabs (MA, USA). AnyFusion and Pfu polymerase were obtained from Genenmed (Seoul, Korea). Ψ5 Ad5 was described previously^25^. Chemical reagents were obtained from Sigma (MO, USA). Dulbecco's modified Eagle's medium and fetal bovine serum (FBS) were purchased from Welgene (Gyeongsangbuk-do, Korea) and CellSera (NSW, Australia), respectively. Chemically competent XL-1 Blue and DH10b cells were purchased from RBC (Taipei, Taiwan). The human *dystrophin* gene, the codon-optimized human *huntingtin* gene, and miRs were synthesized by GenScript (NJ, USA). Other PCR primers and synthetic oligos were obtained from Cosmogenetech (Seoul, Korea). Nucleotide sequence analysis was also performed by Cosmogenetech. The T-Blunt PCR cloning kit and LaboPass Tissue Genomic DNA Isolation Kit were purchased from SolGent (Daejeon, Korea) and Cosmogenetech, respectively. Q Sepharose XL and Chelating Sepharose FF resin for column chromatography were obtained from GE Healthcare (IL, USA). The Vivaspin Turbo ultrafiltration spin column (100 kDa cut-off) was purchased from Sartorius (Goettingen, Germany). Benzonase was obtained from Merck (Darmstadt, Germany). *Dystrophin*-knockout MDX (C57BL/10ScSn-Dmdmdx/J) and wild-type mice (C57BL/10J) were obtained from the Jackson Laboratory (ME, USA). A dystrophin antibody (ab15277) and a huntingtin antibody (sc-47757) were purchased from Abcam (Cambridge, UK) and Santa Cruz Biotechnology (CA, USA), respectively. A β-actin antibody (Abc-2002) was obtained from AbClon (Seoul, Korea). SuperSignal West Pico Chemiluminescent Substrate solution was purchased from Fisher Scientific (NH, USA). HEK293T and HEK293 cells were obtained from ATCC (VA, USA). For the cloning and engineering of DNA sequences, standard DNA manipulation techniques were employed.

### Construction of the pBest cloning shuttle plasmid

The DNA fragment containing the Kan^r^-ColE1 region was prepared by PCR using the pGT2 plasmid as a template and the following primer set: F: 5′-GGGCCAAGGATCTGATGGCGCAGGGGA-3′ and R: 5′-CTTGGCCGCAGCGGCCGAGCAAAAGGCCAGCAAAAGGCCA-3′. A DNA fragment encompassing the 5′ homologous stretch, 5′ inverted terminal repeat (ITR), Ψ5 packaging signal, CMV promoter, a multi-cloning site (MCS), an SV40 poly(A) signal and the 3′ homologous stretch (Fig. S1f) was chemically synthesized by GenScript. This synthetic DNA included an *Sfi*I site at the 5′ end and an *Xcm*I site at the 3′ site. The PCR product and synthetic DNA were ligated together using the *Sfi*I and *Xcm*I restriction sites to generate the pBest plasmid.

### Construction of the pAdBest_dITR helper plasmid

To construct Ψ5-Left-Arm-1 (Fig. S1b), a DNA fragment containing the Kan^r^-ColE1 region was prepared as described for pBest using the following primer set: F: 5′-CCCGGATCCGCAGTGGGCTTACATGGCGATAGC-3′ and R: 5′-CCCGTATACATCGATTTAATTAAGAGCAAAAGGCCAGC-3′. The PCR product was digested with *Bam*HI/*Bst*Z17I and ligated with the *Bst*Z17I–*Bam*HI fragment of the Ψ5 genome. Then, the “asterisk to BstZ17I” DNA fragment (Fig. S1a) was prepared by PCR and inserted into Ψ5-Left-Arm-1 to produce Ψ5-Left-Arm-2. In Ψ5-Left-Arm-2, nucleotides 1–3133 (portion of the 5′ ITR and Ψ packaging signal) of Ad5 (GenBank AC_000008) are deleted. Similarly, to construct Ψ5-Right-Arm-1 (Fig. S1c), the DNA fragment containing the Kan^r^-ColE1 region was prepared by PCR using the following primer set: F: 5′-TTAATTAAGCAGTGGGCTTACATGGCGATAGC-3′ and R: 5′-CCCGGATCCATCGATTTAATTAAGAGCAAAAGGCCAGC-3′. The PCR product was digested with *Bam*HI and ligated with the DNA fragment corresponding to the *Bam*HI-3′ ITR of the Ψ5 genome. The partial remaining E3 region was completely deleted to reduce the genome size of the adenovirus by overlapping PCR using the unique *Spe*I (27,082) and *Nde*I (31,089) restriction sites. The resultant Ψ5-Right-Arm-2 construct harbors a deletion corresponding to nucleotides 27,864–31,000 of Ad5. Thereafter, the *Cla*I–*Bam*HI viral DNA was cleaved out from Ψ5-Left-Arm-2 and ligated to Ψ5-Right-Arm-2 to construct pAdBest (Fig. S1d). Removal of the 3′ ITR from pAdBest was carried out via the following steps: pAdBest was first cleaved with *Cla*I/*Eco*RI, and a smaller fragment was isolated and ligated with a synthetic adaptor containing *Cla*I and *Eco*RI, resulting in pAdBest_EcoR_Cla. Subsequently, pAdBest_EcoR_Cla was cut with *Avr*II/*Rsr*II, and a larger fragment was isolated. Using the primer sets Avr_F: 5′-CTGCCTAGGCAAAATAGCACCC-3′ and Avr_dITR_R: 5′-AACGATGTAAGTTTTAGGGCGGAGTAACTTGTATG-3′ and Avr-dITR_F: 5′-GCCCTAAAACTTACATCGTTAATTAAGCAGTGGGC-3′ and Rsr_R: 5′-TAGCGGTCCGCCACACCCAGCC-3′ and pAdBest as a template, overlapping PCR was performed. The PCR product digested with *Avr*II/*Rsr*II was ligated with the larger fragment of pAdBest_EcoR_Cla cleaved with *Avr*II/*Rsr*II. This ligation produced pAdBest_EcoR_Cla_dITR with complete removal of the 3′ ITR from pAdBest. Then, the *Cla*I–*Eco*RI fragment of pAdBest was ligated back to the corresponding sites of pAdBest_EcoR_Cla_dITR to construct pAdBest_dITR (Fig. S1e).

### Construction of the pGLAd genome plasmid

Using the primer set F: 5′-GGTTGGCGCGCCCTACGTCACCCGCCCCGTTCCCAC-3′ and R: 5′-CCCGAGCTCAAACTACATAAGACCCCCACCTTAT-3′ and pAdBest as a template, PCR was performed. The resultant PCR product was cut with *Sac*I/*Asc*I and then ligated with the adapter (Sac_Pst_Avr_Asc_S: 5′-CTTAACCTGCAGATCCTCCTAGGTTTTTGG-3′ and Sac_Pst_Avr_Asc_AS: 5′-CGCGCCAAAAACCTAGGAGGATCTGCAGGTTAAGAGCT-3′) to produce pAdBestGL1 (Fig. S2a). To remove the unique *Cla*I site from pAdBestGL1, pAdBestGL1 was cleaved with *Cla*I, filled-in with Klenow, and then self-ligated, which resulted in pAdBestGL1_dCla. Both pAdBestGL1 and pAdBestGL1_dCla were digested with *Sac*I/*Avr*II and ligated with a synthetic *Hpa*I-site-containing adaptor (Fig. S2b), which generated pAdBestGL2_wtCla and pAdBestGL2, respectively. Then, *Hpa*I-cut pAdBestGL2 was mixed with *Hin*dIII-digested lambda phage DNA (arrowhead, Fig. S2c) and subjected to in vitro homologous annealing (iHoA) to construct pAdBestGL3. pAdBestGL3 was cleaved with *Sac*I/*Apa*I or *Apa*I/*Avr*II and ligated with a *Sac*I/*Apa*I adaptor (Fig. S2d) or an *Apa*I/*Avr*II adaptor (Fig. S2e) to produce pAdBestGL4_3H and pAdBestGL4_5H, respectively. pAdBest4_3H_2dCla was constructed via the consecutive removal of the *Cla*I sites from pAdBestGL4_3H, in which pAdBestGL4_3H was cleaved with *Cla*I, filled-in with Klenow, self-ligated and transformed into Dam−/− bacterial cells. The resultant pAdBestGL4_3H_dCla construct was cut again with *Cla*I, filled-in with Klenow and self-ligated to generate pAdBestGL4_3H_2dCla. Similarly, pAdBestGL4_5H was digested with *Cla*I, filled-in with Klenow and self-ligated to produce pAdBestGL4_5H_dCla. Then, the *Sac*I–*Apa*I fragment (5′ half portion) of pAdBestGL4_5H_dCla was transferred to *Sac*I/*Apa*I-cleaved pAdBestGL4_3H_2dCla to construct pAdBestGL5 (Fig. S2f). To produce pAdBestGL (Fig. S2g), pAdBestGL2_wtCla was cut with *Hpa*I, mixed with *Sac*I/*Avr*II-digested pAdBestGL5 (Fig. S2f) and subjected to iHoA. Then, pAdBestGL5 was cleaved with *Apa*I/*Nsi*I and ligated with the genomic PCR-prepared SMAR element (F: 5′-TCTGGGCCCAAATAAACTTATAAATTGTGAGAG-3′ and R: 5′-CCCATGCATATATTTAAAGAAAAAAAAATTGTA-3′) to finally construct pGLAd (Fig. S2h).

### In vitro homologous annealing

iHoA was performed using AnyFusion. The entire procedure followed the manufacturer’s instructions with slight modification: incubation was performed for 10 min at 55 °C, followed by further incubation for 20 min at room temperature. Then, the reaction mixture was transformed into chemically competent XL-1 Blue or DH10b cells and spread over an antibiotic-containing agar plate.

### Generation of the GLAd.LacZ and GLAd3.LacZ viruses

The pGLAd_LacZ or pGLAd3_LacZ plasmid (10 µg) was cut with the *Pac*I restriction enzyme, and *Pac*I activity was heat-inactivated. This *Pac*I-linearized pGLAd_LacZ or pGLAd3_LacZ genome plasmid and 30 µg of the pAdBest_dITR helper plasmid were co-transfected into HEK293T cells plated in a 100 mm culture dish using the calcium phosphate precipitation method. After 6 h of incubation, the culture medium (10% FBS) was changed to fresh medium (5% FBS). Forty-eight hours later, the transfected cells and media were harvested (in this step, the culture medium was saved as the viral medium), and the cells were resuspended in 1 ml of fresh culture medium (5% FBS) and disrupted through three cycles of freezing and thawing (the cleared lysate was referred to as the viral lysate). The viral lysate was harvested and used to infect HEK293 cells (recombinant GLAd.LacZ and GLA3.LaZ viruses cannot induce lytic cell death in treated HEK293 cells because the GLAd virus alone cannot be amplified in HEK293 cells). Forty-eight hours after treatment, the cells were stained for LacZ expression.

### LacZ staining

The culture medium was removed from treated cells, and the cells were fixed with fresh fixation solution (2% formaldehyde/methanol and 0.1% glutaraldehyde in phosphate-buffered saline (PBS)) for 2 min at room temperature. After two careful washes with PBS, the cells were incubated with staining solution (1 mg/ml X-gal, 2 mM MgCl_2_, 5 mM K ferri-cyanide, and 5 mM K ferro-cyanide in PBS) at 37 °C until LacZ staining was evident.

### Purification of genomic DNA from the mouse tail

Genomic DNA was purified from the tail of a C57BL/6J mouse using the LaboPass Tissue Genomic DNA Isolation Kit. The entire procedure followed the manufacturer’s instructions with slight modification. Briefly, the mouse tail (2 × 2 mm piece) was incubated with lysis buffer containing proteinase K until the tissue was completely lysed. The sample was mixed with 100% ethanol and passed through a mini spin column. Bound genomic DNA was thoroughly washed with two different wash buffers and eluted with distilled water.

### Cloning of the mouse E-cadherin intron 2 region as a pGLAd3 genome backbone

Genomic DNA purified from the mouse tail was used as a PCR template. The primer sets and sequences are described in Supplementary Table 1. PCR amplification of the F1, F2, F3, F4, or F5 fragment was performed for 45 cycles (30 s at 95 °C, 30 s at 60–65 °C, 4 min at 72 °C) with each primer set using Pfu polymerase. Each PCR product was cloned into the T-Blunt vector of the T-Blunt PCR cloning kit. The resultant T-F1, T-F2, T-F3, T-F4, and T-F5 products (Fig. S6b) were sequenced for verification.

### Construction of the pGLAd3 genome plasmid

Using genomic DNA from the mouse tail as a PCR template and the primer sets N-F/N-R and C-F/C-R (Table S1), overlapping PCR was carried out for 50 cycles (30 s at 95 °C, 30 s at 62 °C, 60 s at 72 °C). The resultant NC fragment PCR product, containing sites for restriction enzymes such as *Bsp*EI, *Xho*I, and *Nsi*I, was subjected to iHoA with pGLAd cut with SacI/AvrII (Fig. S6b) to produce pGLAd_NC. Next, pGLAd_NC was cleaved with *Nsi*I/*Xho*I and ligated with the annealed synthetic Nsi–Xho fragment (Table S1) to construct pGLAd_NC_PU, which was the final recipient harboring the F12345 fragment. In parallel, T-F1_dPac was generated by removing the unique *Pac*I site of T-F1 by cutting with *Pac*I, blunting with Klenow and self-ligation. T-F2_SMAR was prepared following the ligation of *Sfi*I/*Sal*I-cleaved T-F2 with the SMAR element prepared by PCR using the SMAR-F/SMAR-R primers (Table S1) and pGLAd as a template. Then, T-F12 was generated by transferring the *Not*I/*Rsr*II-cut T-F1_dPac fragment to *Not*I/*Rsr*II-cleaved T-F2_SMAR. The construction of F-T34 was carried out following the transfer of the T-F4 fragment, which was processed with cutting with PvuI, blunting with Klenow, heat-inactivating and additionally cutting with *Pci*I, to the T-F3 that was obtained through processing via cutting with *Spe*I, blunting with Klenow, heat-inactivating and additionally cutting with PciI. T-F345 was produced by ligating the *Mlu*I–*Xba*I fragment of T-F5 with T-F34 cut with *Spe*I/*Mlu*I (the *Spe*I site can be ligated to the *Xba*I site). T-F12345 was generated by transferring the *Sal*I–*Rsr*II fragment of T-F12 to the T-F345 cut with *Sal*I/*Rsr*II. Ultimately, pGLAd3 was constructed utilizing iHoA between *Pst*I-cut pGLAd_NC_PU and *Xho*I-cut T-F12345. In every step of the construction procedure, sequencing was performed for verification.

### Construction of the pBest4 cloning shuttle plasmid

The pCAG portion of the plasmid was prepared by PCR using a following primer set: F: 5′-GTTATTAATAGTAATCAATTACG-3′ and R: 5′-CTTGGGTCTCCCTATCGCCCGCCGCGCGCTTCGCTTTTTATAGG-3′, and then cut with *Ase*I and *Bsa*I. The β-globin intron region was also prepared by PCR with the following primers: F: 5′-GGCGATAGGGAGACCCAAGCTGGTGAGTTTGGGGACCC-3′ and R: 5′-GGGAAGCTTGGGTCCCCTGTAGGAAAAAGAAGAAGGCATGAAC-3′, and cut with *Bsa*I and *Hin*dIII. To construct the pBest4 shuttle plasmid, the pBest cleaved with *Ase*I/*Hin*dIII was ligated with the prepared PCR products.

### Plaque assay

HEK293 cells (80–90% confluency) plated in a 100 mm culture dish were treated with Ad.LacZ (first-generation Ad as a positive control; 1 × 10^3^ infectious viral particles), GLAd3.LacZ (1.12 × 10^7^–1.80 × 10^7^ BFU) or the cell lysate prepared from HEK293T cells transfected with the helper plasmid alone (Table 1). Twenty-four hours later, the culture medium was aspirated, carefully overlaid with 12 ml of culture medium containing sterile agarose (0.3%) and incubated in a CO_2_ incubator for 10–15 days. A 10 ml aliquot of the diluted MTT solution was added to the agarose layer and incubated for 5 h. Viral plaques were counted on a light box.

**Table 1:** GLAd yield and other viruses generated during GLAd production

		GLAd yield^a^ (total BFU)	Other viruses^b,c^
Helper plasmid + pGLAd3_LacZ genome^d^	GLAd3.LacZ #1	1.20 × 10^7^	None
	GLAd3.LacZ #2	1.12 × 10^7^	None
	GLAd3.LacZ #3	1.80 × 10^7^	None
Helper plasmid^d^	#1	N/A	None
	#2	N/A	None
	#3	N/A	None
Ad.LacZ^e^ (1 × 10^3^ used)			~10^3^

*BFU* blue-forming units, *N/A* not applicable

^a^Determined by LacZ staining (total BFU)

^b^Adenovirus and/or RCA

^c^Determined by plaque assay

^d^Transfected into HEK293T cells plated on 100 mm culture dish

^e^Positive control for plaque assay

### Construction of the pAd5pTP expression plasmid

The Ad5 pTP gene was prepared by PCR using the Ad Ψ5 DNA as a template and the following primer set: F: 5′-GGGAAGCTTACCATGGCCTTGAGCGTCAACGATTGCGCGCGCCTGACC-3′, underlined is *Hin*dIII site; R: 5′-GGCGAATTCCTAAAAGCGGTGACGCGGGCGAGCC-3′, underlined is *Eco*RI site. PCR was carried out for 40 cycles (30 s at 95 °C, 30 s at 60 °C, 120 s at 72 °C). The resultant PCR product was digested with *Hin*dIII and *Eco*RI and cloned into the pLV_XL plasmid prepared from pLV_VSVG_XL following cleavage with *Hin*dIII and *Eco*RI.

### Large-scale production of recombinant GLAd

To produce recombinant GLAd at a large scale, continuous amplification was employed as demonstrated in the standard method for conventional GLAd production. In detail, HEK293T cells (50–70% confluency) plated in a 100 mm dish were transfected with a mixture of 10 µg of pGLAd3_LacZ (cut with *Pac*I and heat-inactivated), 30 µg of pAdBest_dITR and 2.5 µg of pAd5pTP using the calcium phosphate precipitation method. After 6 h of incubation, the culture medium (10% FBS) was changed to fresh medium (5% FBS). Forty-eight hours later, the transfected cells were harvested, resuspended in 1 ml of fresh culture medium (5% FBS) and disrupted through three cycles of freezing and thawing to rescue the GLAd3.LacZ virus (P0 seed GLAd). For the first round of amplification (P1), HEK293T cells (50–70% confluency) plated in a 100 mm dish were transfected with a mixture of 45 µg of pAdBest_dITR and 3.75 µg of pAd5pTP using the calcium phosphate precipitation method. After 6 h of incubation, the culture medium (10% FBS) was changed to fresh medium (5% FBS) containing P0 seed GLAd. Forty-eight hours later, the transfected cells and media were harvested (in this step, the culture medium was saved as the viral medium), and the cells were resuspended with 1 ml of fresh culture medium (5% FBS) and disrupted through three cycles of freezing and thawing (the cleared lysate was referred to as the viral lysate). The viral lysate was harvested and combined with the viral medium (total 10 ml) and used to infect HEK293T cells (plated in 10 × 150 mm dishes) for the next round of amplification (P2) (see Fig. 3). Additional rounds of amplification (P3, P4, P5 and so on) can be carried out to increase the GLAd production scale. The flow chart for each round of amplification, the obtained viral titers, the number of plates needed and the required amounts of the helper and pAd5pTP plasmids are described in detail in Fig. 3.

### Amplification of adenovirus and RCA contaminants generated during GLAd production

Unlike GLAd, adenovirus and RCA can replicate in HEK293 cells. Thus, HEK293 cells were infected with Ad.LacZ (positive control) or GLAd3.LacZ (P3, 3 × 10^9^ BFU; P4 or P5, 1 × 10^8^ BFU), and the potential contaminant adenovirus and RCA in GLAd3.LacZ were allowed to replicate during one (P4 or P5), approximately two (positive control), or three (P3) rounds of amplification. Because an MOI of GLAd3.LacZ that is too high, resulting in overexpression of LacZ, can kill infected cells, two 150 mm dishes were used for the initial infection of GLAd3.LacZ (P3, 3 × 10^9^ BFU). Benzonase was employed to degrade the helper plasmid that was continuously used for the preparation of P3, P4, or P5 GLAd3.LacZ (the helper plasmid contains the target gene for PCR-based analysis; the viral DNA packaged into the capsid shell is resistant to Benzonase). To examine its effects on the infectivity of adenovirus and RCA, Benzonase treatment was also applied to the positive control (Ad.LacZ). For each round of amplification, cells were infected with the corresponding virus and then harvested after 72 h. These cells were resuspended in a volume of 1 ml (for a 100 mm dish) or 2.5 ml (for each 150 mm dish) and disrupted via three cycles of freezing and thawing (the cleared lysate was referred to as the viral lysate). Each viral lysate was used for further amplifications, and the cell lysate and culture medium were finally harvested together. During these amplification processes, CPE was examined under a microscope (for the entire workflow, see Fig. 4a, c).

### Analysis of adenovirus and RCA contaminants by PCR

The serially amplified samples from HEK293 cells (Fig. 4a, c) were first heat-treated for 10 min at 95 °C (if this step is omitted, endogenous cellular DNase degrades the spiked Ad5 DNA) and then analyzed by PCR for N-terminal DNA of the *fiber* gene, which is present in both adenovirus and RCA but not in the GLAd genome or HEK293 cells. Ad5 DNA (10 pg, Ad Ψ5 DNA) was used as a positive control for PCR. Ad.LacZ samples were subjected to PCR before or after 100× dilution, in which only 500 virus particles are contained in the sample. GLAd3.LacZ samples were PCR-amplified in the presence or absence of spiked Ad5 DNA (10 pg). PCR was carried out with Pfu polymerase and the following primer set: F: 5′-CGCGCAAGACCGTCTGAAGATACC-3′ and R: 5′-GGCCTGATGTTTGCAGGGCTAGC-3′, for 40 cycles (30 s at 95 °C, 30 s at 60 °C, 30 s at 72 °C), and the results were analyzed by agarose gel electrophoresis (Fig. 4b, d).

### Construction of the pGLAd4 genome plasmid

The pGLAd3 contains two *Bss*HII restriction sites (Fig. S8). Treatment of pGLAd3 with *Bss*HII and self-ligation resulted in pGLAd4, which decreased the length of the plasmid from 26,597 bp (pGLAd3) to 16,392 bp (pGLAd4). After completion, the *Bss*HII site was sequenced for verification.

### Construction of the *huntingtin* mshR expression plasmids

The template for mshR expression, which was confirmed to be fully functional, was described previously^26,27^. Based on these conserved sequence and structural characteristics, the corresponding DNAs were synthesized (Table S2) and cloned into the pGT2 plasmid using the *Bam*HI and *Eco*RI sites.

### Knockdown of endogenous huntingtin expression by mshRs

HEK293T cells plated in a 100 mm culture dish were transfected with 15 µg of pGT2, pGT2-mshR1, pGT2-mshR2, or pGT2-mshR3. Forty-eight hours later, untreated control and transfected cells were harvested and subjected to western blotting analysis for endogenous huntingtin expression.

### Western blotting

Whole-cell lysates prepared using RIPA lysis buffer were resolved in an SDS gel and transferred to a nitrocellulose membrane. The membrane was blocked with Blotto A solution (TBST, 5% milk) for 1 h at room temperature and further incubated with Blotto B solution (TBST, 1% milk) containing the primary antibody (1:500–1000) at 4 °C overnight. After one 5 min wash with TBST (TBS, 0.05% Tween-20; TBS (Tris-buffered saline): 10 mM Tris-Cl (pH 8.0), 150 mM NaCl), the membrane was incubated with an HRP-conjugated secondary antibody (1:5000–100,000) in Blotto B solution for 2 h at room temperature. Following two washes (5 min each) with TBST, the membrane was briefly treated with the SuperSignal West Pico Chemiluminescent Substrate solution, and the resulting image was analyzed with a Bio Imaging System.

### Construction of the pGLAd4_HTTmshR1/3

HTTmshR1 and HTTmshR3 encompassing pCMV, mshR and BGHpA (Fig. 5d) were prepared by PCR using appropriate primer sets (Table S3) and pGT2-mshR1 or pGT-mshR3 as a template (Fig. 5c, d). To construct pGLAd4_HTTmshR1/3, the resultant HTTmshR1 and HTTmshR3 constructs were subjected to iHoA with Acc65I-cut pGLAd4 and PciI-cut pGLAd4, respectively (Fig. 5f). After completion, the regions adjacent to the *Acc*65I and *PciI* sites were sequenced for verification.

### Construction of pGLAd4_coHTT.HTTmshR1/3 and pGLAd4_coHTT(R).HTTmshR1/3

The full-length codon-optimized synthetic *huntingtin* gene (9.4 kb) containing Q22 was cloned into the pBest4 shuttle plasmid in different orientations. In an approach previously demonstrated as a standard procedure, pBest4_coHTT and pBest4_coHTT(R) were cut with *Pme*I and subjected to iHoA with ClaI-cleaved pGLAd4_HTTmshR1/3. This iHoA process produced the pGLAd4_coHTT.HTTmshR1/3 or pGLAd4_coHTT(R).HTTmshR1/3 construct. Successful completion was verified by sequencing.

### Production of recombinant GLAd4.coHTT.HTTmshR1/3 and GLAd4.coHTT(R).HTTmshR1/3 viruses

The same procedure used for producing the recombinant GLAd.LacZ and GLAd3.LacZ viruses was applied.

### Purification of recombinant GLAd4.Dys virus

The recombinant GLAd4.Dys virus was produced as described (Fig. 3) and purified from a P3 preparation. For purification, two column-based chromatography methods were employed as reported previously^28^, with slight modification. Briefly, the cell lysate and the harvested culture medium were combined (P3), treated with Benzonase, filtered and subjected to Q Sepharose column chromatography as a first step of purification. After washing, the bound virus was eluted and diluted with buffer and loaded onto a Zn-chelated chromatography column. The column was thoroughly washed, and the bound virus was eluted. Buffer change to a formulation buffer and concentration of virus were simultaneously carried out utilizing Vivaspin Turbo ultrafiltration spin column. The formulation buffer was described previously^29^.

### Determination of GLAd4.Dys viral particles

The purified recombinant GLAd4.Dys virus was serially diluted using virus lysis buffer (0.1% SDS, 10 mM Tris-Cl (pH 7.4), 1 mM EDTA) and incubated for 10 min at 56 °C with gentle shaking. Then, the OD_260_ was determined and subjected to the calculation of the virus particle concentration using the equation: (OD_260_) × (virus dilution factor) × (1.1 × 10^12^) = virus particles (VP/ml). In this calculation, the blank solution consisted of the virus lysis buffer and the virus formulation buffer. The extinction coefficient was used as established: OD_260_ unit = 1.1 × 10^12^ virus particles/ml.

### Animal study

All animal experiments were conducted according to the protocol (KNU2018-0134) approved by the Institutional Animal Use and Care Committee (IAUCC) of Kyungpook National University (Daegu, Korea). Eight-week-old male wild-type control mice (C57BL/10J) and *dystrophin*-knockout MDX mice (C57BL/10ScSn-Dmdmdx/J) were housed under 12 h light–dark cycles and given water freely in accordance with the Kyungpook National University Animal Facility regulations. The focal gastrocnemius muscles of the MDX mice were injected intramuscularly with PBS (*n* = 3) or with 50 μl of recombinant GLAd4.Dys virus (4 × 10^10^ particles) (*n* = 3). Four weeks later, muscle tissues were biopsied and subjected to analysis.

### Immunofluorescence staining

The immunofluorescence staining of dystrophin was performed as described previously^30^ with slight modification. Biopsied muscle tissues were fixed at 4 °C overnight with freshly prepared 4% PFA in PBS. Then, the tissues were incubated with 5% sucrose in PBS at 4 °C for 6 h and further incubated with 20% sucrose in PBS at 4 °C overnight. The processed tissues were embedded in OCT, frozen by dipping in liquid nitrogen-chilled isopentane, and stored at −70 °C. Four-micron-thick cross-sections were produced, then placed in PBS for 10 min and washed with PBST (0.1% Triton X-100 in PBS) three times (10 min each) at room temperature. The tissue sections were blocked with 10% horse serum in PBST at 4 °C overnight, incubated with a dystrophin antibody (1:100 in blocking buffer) at 4 °C overnight, and then washed with PBST three times (10 min each) at room temperature. Finally, the tissue sections were incubated with a TRITC-conjugated secondary antibody (1:100 in blocking buffer) at 4 °C overnight and washed with PBST three times (10 min each) at room temperature. The stained tissue sections were covered with mounting medium containing DAPI and analyzed under a confocal microscope.

## Results

### Construction of the pAdBest_dITR helper plasmid and pGLAd genomic plasmid and their use for producing GLAd

**Fig. 1:**
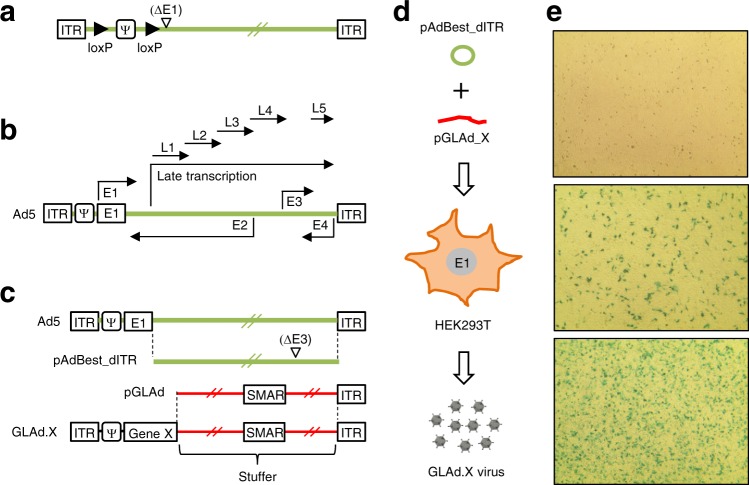
Construction of the pAdBest_dITR helper plasmid and the pGLAd genome plasmid and their use for producing GLAd. **a** Structural characteristics of the most commonly used helper adenovirus. The black arrowheads indicate loxP sites. **b** Ad5 genome structure and transcription units. E and L indicate the early gene and the late gene, respectively. The arrows indicate the orientation of transcription units. **c** Schematic illustration and comparison of Ad5, pAdBest_dITR helper plasmid, pGLAd genome plasmid, and GLAd genome structures. SMAR indicates the scaffold/matrix attachment element. Colors indicate the origin of the DNA backbones. **d** Schematic illustration of GLAd production. Co-transfection of the pAdBest_dITR helper plasmid and the *Pac*I-linearized recombinant pGLAd_X genome plasmid into HEK293T cells produces recombinant GLAd.X virus. **e** LacZ staining of HEK293 cells infected with the GLAd.LacZ virus. At 48 h after the infection of HEK293 cells with the GLAd.LacZ virus, the cells were subjected to LacZ staining. Upper: untreated control (background dots are X-gal crystals, not stained cells); middle: 50 μl out of 10 ml viral medium was used for infection; bottom: 5 μl out of 1 ml viral lysate was used for infection

Currently, the most commonly used helper adenovirus contains loxP sites flanking the Ψ packaging signal^21,22^ (Fig. 1a). Its structural characteristics allow this particular adenovirus to efficiently produce recombinant GLAd with reduced contamination of adenovirus in Cre-expressing packaging cells^21–24^. Nevertheless, contamination of helper adenovirus is unavoidable even in this sophisticated production system. Moreover, homologous recombination between the helper adenovirus and the E1 region present in packaging cells generates RCA^21^, although RCA has not been intensively analyzed in this system^21–24^. We hypothesized that the conversion of the helper adenovirus into a helper plasmid following the deletion of the region involved in homologous recombination might prevent the generation of both adenovirus and RCA contaminants in GLAd preparations. To achieve this goal, we attempted to generate our own helper plasmid that supplies all viral proteins for GLAd packaging but is securely unpackageable into active viral particles, resulting in no generation of RCA.

Our helper adenovirus-free recombinant GLAd production system requires two independent plasmids: one serving as a helper for GLAd packaging and the other as the GLAd genome. As our helper plasmid, we constructed pAdBest_dITR (~31 kb) (Fig. S1). Based on the overlapping transcription units and multiple transcripts governed by a single promoter (Fig. 1b), we manipulated the Ψ5 genome^25^, a derivative of Ad5 (Fig. S1a), and obtained this helper plasmid. Our helper plasmid contains neither ITRs nor Ψ packaging signal (Fig. 1c), both of which are essential for virus packaging; thus, this plasmid only serves as a helper but is securely unpackageable into active viral particles. During this manipulation, the 5′ ITR and Ψ packaging signal were transferred to pBest, a shuttle plasmid (Fig. S1f) that is used for transgene cloning.

As our GLAd genome plasmid, we constructed pGLAd (Fig. 1c). Our pGLAd is composed of stuffer DNA from lambda phage and only the 3′ ITR of Ad5. The scaffold matrix attachment (SMAR) element^31^ was used to stabilize the GLAd genome in cells while enhancing transgene expression (for entire construction processes, see Fig. S2).

Generally, it is a tedious process to insert a transgene into a large plasmid such as the pGLAd genome plasmid (~27 kb). To facilitate this process, we added short homologous stretches to both pBest and pGLAd when constructing these plasmids. These homologous stretches expedited the transfer of the transgene expression cassette from pBest to pGLAd. As an example of this process, the LacZ gene was first cloned into pBest, which was then linearized with the rare-cutting enzyme *Pme*I (Fig. S3a). Simultaneously, pGLAd was cut at the unique *Cla*I site. When linearized pBest_LacZ and linearized pGLAd were mixed and treated with AnyFusion, a nuclease that can convert double-stranded DNA to single-stranded DNA, both linearized homologous regions were efficiently annealed in vitro (the entire process is referred to as “iHoA” for in vitro homologous annealing) (Fig. S3b). The length of the homologous stretches annealed in vitro results in sufficient stability to transform bacterial cells. The resultant recombinant pGLAd_LacZ plasmid recovered both the ITRs and the Ψ packaging signal, and the ITRs were flanked by rare-cutting *Pac*I restriction sites (Fig. S3c). The entire process is quick and efficient in the transfer of a transgene expression cassette to the pGLAd genome plasmid. Moreover, in the transformation step, the bacterial cells containing the correct pGLAd_LacZ plasmid formed colonies that were markedly smaller in size than those of other undesired clones (Fig. S4a). This visually observable characteristic accelerated the screening process (Fig. S4b–d). After the completion of this process, the junctions of the homologous stretches of the resultant recombinant plasmid were sequenced. The nucleotide sequences perfectly matched the homologous stretches (Fig. S5), indicating that the iHoA process operated as designed.

After the construction of both the pAdBest_dITR helper plasmid and the recombinant pGLAd_LacZ genome plasmid, we tested our two-plasmid-based recombinant GLAd production system. We co-transfected HEK293T cells with the pAdBest_dITR helper plasmid and the PacI-linearized pGLAd_LacZ genome (Fig. 1d, e). Co-transfected HEK293T cells successfully produced recombinant GLAd.LacZ virus (Fig. 1e), demonstrating that all the constructs and processes involved in the production of recombinant GLAd worked properly as designed.

### Construction of the pGLAd3, a new genome plasmid

**Fig. 2:**
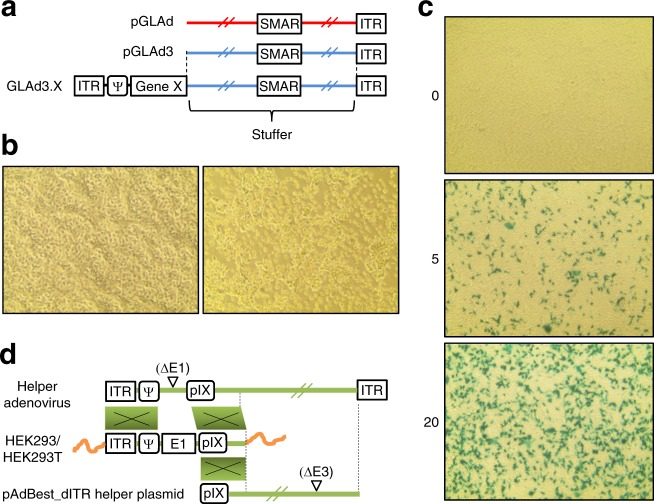
Construction of the pGLAd3, a new genome plasmid, and its use for producing GLAd. **a** Schematic illustration and comparison of pGLAd and pGLAd3 genome plasmid and corresponding GLAd. Colors show the origin of the DNA backbones. **b** Cytopathic effect (CPE) observed during the production of recombinant GLAd3.LacZ virus. The pAdBest_dITR helper plasmid and the PacI-cut pGLAd3_LacZ genome plasmid were co-transfected into HEK293T cells. Forty-eight hours later, untreated control (left) and transfected cells (right) were photographed under the microscope. **c** LacZ staining of HEK293 cells infected with the GLAd3.LacZ virus. At 48 h after the infection of HEK293 cells with the GLAd3.LacZ virus, the cells were subjected to LacZ staining. The numbers indicate the viral lysate volume (0, 5, or 20 μl) used for infection from 1 ml viral lysate prepared from a 100 mm culture dish. **d** Homologous regions identified in the helper adenovirus, E1-expressing packaging cells and our pAdBest_dITR helper plasmid. Homologous recombination can occur in colored boxed regions. RCA generation requires two homologous recombination events, through which E1 is transferred from the packaging cell to the helper adenovirus

Interestingly, the nature of the stuffer in GLAd has been shown to negatively affect transgene expression. In particular, a lambda DNA stuffer showed this undesirable characteristic, although this finding is controversial^32,33^. Nevertheless, under the initial conditions, we utilized lambda DNA to quickly examine the validity of the designed plasmid construction schemes and to determine whether our helper plasmid-based GLAd production system would function properly as expected. Since our GLAd production system operated as designed, we prepared a new stuffer to reduce concern about the decreased expression of transgenes in the presence of lambda stuffer. We cloned genomic fragments from the mouse E-cadherin intron 2 region^34^ (Fig. S6a) and generated pGLAd3 as a new GLAd genome plasmid (Fig. 2a, Fig. S6b). We also constructed another cloning shuttle plasmid, pBest4 (Fig. S6c), which was upgraded with a strong CAG promoter and an intron. Similar to the original system, this new system tested with the LacZ gene also effectively produced recombinant GLAd3.LacZ virus (Table 1, Fig. 2b, c) with a cytopathic effect (CPE) (Fig. 2b), indicating that all the elements and processes, such as pCAG, the intron, iHoA, and GLAd packaging, performed correctly as designed. In sharp contrast, HEK293T cells transfected with the pAdBest_dITR helper plasmid alone did not produce any viral particles (Table 1). Additionally, recombinant GLAd produced by our two-plasmid-based system did not contain any other viral species, such as adenovirus or RCA (Table 1). It is clear that our helper plasmid only provides helper function for GLAd packaging but is securely unpackageable into active viral particles. Furthermore, these results indicate that in accordance with its structural characteristics (Fig. 2d), our helper plasmid does not generate RCA (Fig. S7).

### Large-scale GLAd production

**Fig. 3:**
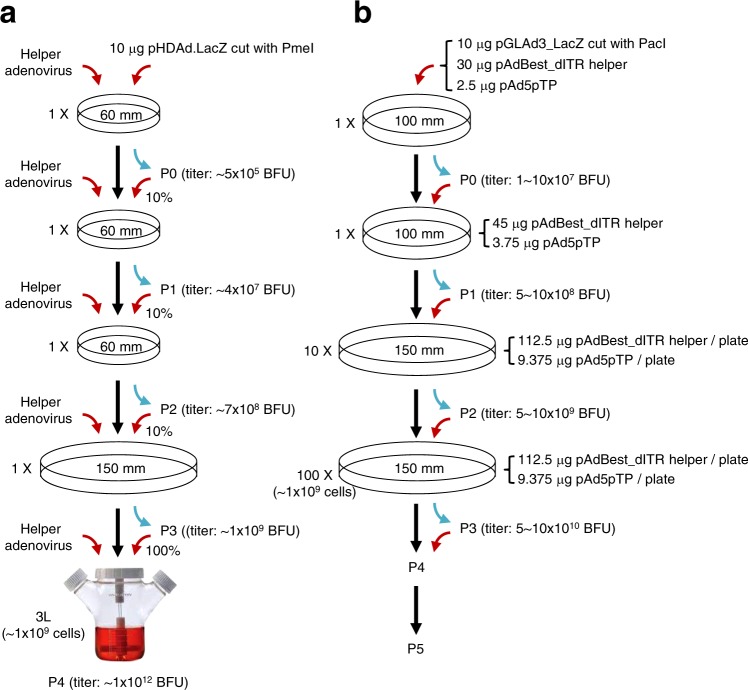
Workflow for large-scale GLAd production. **a** The entire process of the standard conventional GLAd production method. The pHDAd.LacZ is a GLAd genome plasmid containing the LacZ expression cassette. The titer indicates the total infectious GLAd particles (BFU determined by LacZ staining) produced in each round of amplification. **b** The entire process for seed GLAd (P0) rescue, continuous amplification (P1 to P3) and large-scale GLAd production (P3, P4, or P5, depending on scale) is described (for details, see Materials and methods). The titer (BFU) was determined by LacZ staining in each round of amplification

The optimized standard method for conventional large-scale GLAd production involves a serial amplification process^22–24^ (Fig. 3a). Every round of this process requires freshly added helper adenovirus since GLAd can be amplified only in the presence of the helper adenovirus. Seed GLAd is repeatedly amplified over 4–6 rounds prior to the final round of amplification in large-scale cell culture (~1 × 10^9^ cells). This entire amplification process routinely produces ~1 × 10^12^ BFU (~1000 BFU/cell; BFU, blue-forming units determined by LacZ staining)^22^. This relatively high yield of GLAd is attributed to the use of a replicable helper adenovirus that can supply a sufficient amount of viral proteins for GLAd packaging and amplification.

In an attempt to produce GLAd at a large scale utilizing our helper plasmid, we followed the standard amplification procedure established for conventional large-scale GLAd production, although our helper plasmid cannot replicate in GLAd packaging cells. We have already shown successful production of seed GLAd (~2 × 10^7^ BFU/100 mm dish) by transfecting the *Pac*I-cut GLAd genome plasmid and our helper plasmid (Table 1). This seed GLAd was well amplified by our helper plasmid, and its levels were scaled up (data not shown), even though the amplification efficiency was not yet optimized.

Previously, it was shown that the additional expression of adenoviral proteins such as E1A, pTP, and IVa2, either individually or in combination, increases adenovirus production^35–37^. We tested these adenoviral proteins individually and in combination to improve amplification efficiency in GLAd production. The additional expression of only precursor terminal protein (pTP) increased GLAd production (~4-fold) (data not shown). Furthermore, we observed that a greater amount of helper plasmid (45 µg/100 mm dish rather than 30 µg/100 mm dish) resulted in a higher yield in GLAd production (in P1 and thereafter, not in P0; data not shown). For each amplification procedure, the pAdBest_dITR helper plasmid and the pTP-expressing plasmid were co-transfected into 293T packaging cells, similar to the conventional amplification procedure utilizing helper adenovirus. Through optimization, we successfully established a standard procedure for our helper plasmid-based large-scale GLAd production (Fig. 3b). P3 routinely produced 5 × 10^10^– 1 × 10^11^ BFU (50–100 BFU/cell), which is only a 10–20-fold-lower yield compared to the conventional large-scale production method utilizing replicable helper adenovirus^22^ (for example, conventional method: new method = 1–2 × 10^11^ BFU: 1 × 10^10^ BFU). P5 is expected to produce 5 × 10^12^– 1 × 10^13^ BFU from 1 × 10^11^ cells.

### Absence of adenovirus and RCA contaminant generation during GLAd production

Preventing contamination of adenovirus and RCA in GLAd preparation is crucial for considering the clinical application of GLAd. We have demonstrated that GLAd can be produced by the transfection of the *Pac*I-cut GLAd genome plasmid and our helper plasmid, and none of the three independent GLAd preparations contained any adenovirus and/or RCA (Table 1). Nevertheless, we analyzed adenovirus and RCA more intensively again in GLAd preparations (P3, P4, or P5) (Fig. 3) whose scales were large enough and, thus, might be used to produce the clinical materials in the future.

**Fig. 4:**
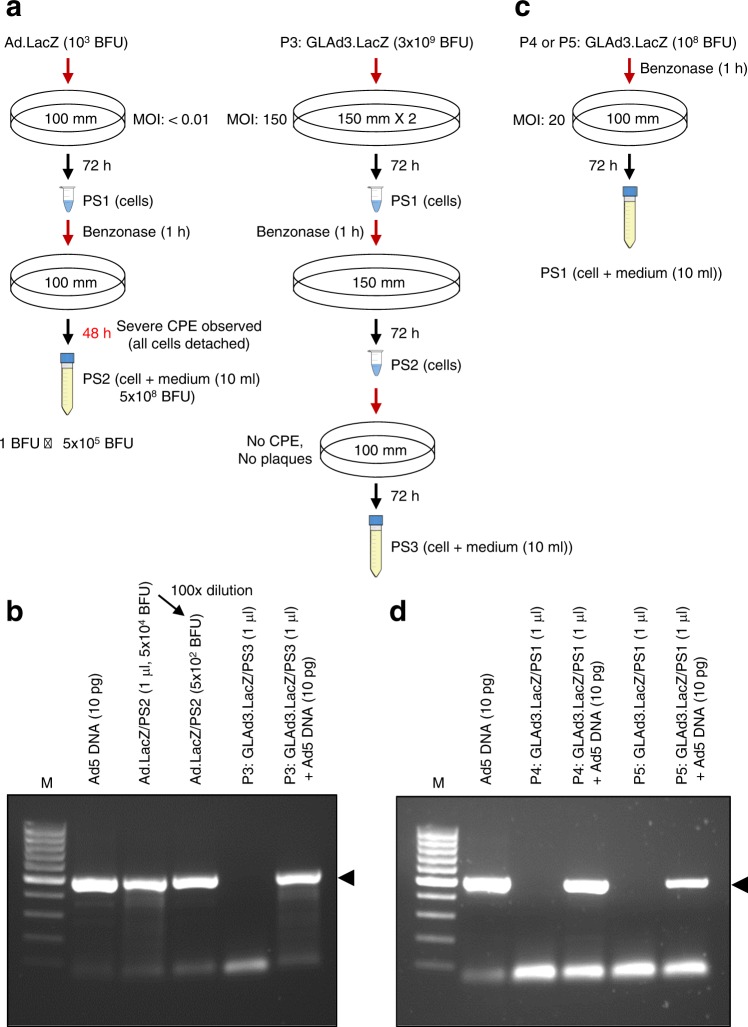
Analysis of adenovirus and RCA contaminants generated during GLAd production. **a** Serial infection of HEK293 cells in 100 or 150 mm culture dishes with Ad.LacZ (positive control) or P3 GLAd3.LacZ (3 × 10^9^ BFU) (see Fig. 3 for production), respectively. Under these conditions, replicable adenovirus and RCA can be amplified. In the positive control, one infectious virus particle resulted in 5 × 10^5^ infectious virus particles (1 BFU to 5 × 10^5^ BFU) within less than two complete rounds of amplification. Benzonase was used to degrade the helper plasmid continuously used for the preparation of P3 GLAd3.LacZ (viral DNA packaged into the capsid shell is resistant to Benzonase). In each round of amplification, the cells were collected and processed to release adenovirus and RCA. In the final round of amplification, both the cells and culture medium were collected and processed for the analysis of adenovirus and RCA. **b** Analysis of adenovirus and RCA in the samples (**a**) by PCR. The samples were analyzed by PCR for the N-terminal DNA of the *fiber* gene, which is present in both the adenovirus and RCA but not in the GLAd genome or HEK293 cells. Ad5 DNA (10 pg) was used as a positive control for PCR. The Ad.LacZ sample was subjected to PCR before or after 100× dilution (in this dilution, only 500 virus particles were present in the sample). The GLAd3.LacZ sample was PCR-amplified without or with spiked Ad5 DNA (10 pg). The arrowhead indicates the target PCR product (484 bp). M is a 100 bp size marker. **c** Infection of HEK293 cells with P4 GLAd3.LacZ or P5 GLAd3.LacZ for the amplification of adenovirus and RCA, as shown in **a**. The prepared samples were subjected to PCR analysis (**d**). **d** Analysis of adenovirus and RCA in samples (**c**) by PCR. PCR was carried out as described in **b**

Adenovirus (Ad.LacZ) used as a positive control was efficiently amplified in HEK293 cells (Fig. 4a). One infectious viral particle (1 BFU) generated ~5 × 10^5^ BFU in less than two complete rounds of amplification. This robust amplification activity of adenovirus caused a severe CPE, which was easily observable with the naked eye. These results suggest that even a single infectious contaminant adenovirus (or RCA) particle in GLAd preparations results in numerous viral particles after a few rounds of amplification. We analyzed P3 GLAd3.LacZ (Fig. 3) containing 3 × 10^9^ BFU (Fig. 4a). We infected HEK293 cells with this GLAd and allowed the amplification of adenovirus and RCA, if any, for three consecutive rounds. These amplification conditions were sufficient for 1 BFU to generate at least 5 × 10^5^ BFU, causing a severe CPE in HEK293 cells in the final round; however, we could not observe any CPE in infected cells. Accordingly, we were unable to detect any adenoviral species (either adenovirus or RCA) in the PCR-based analysis (Fig. 4b). Our data indicate that the preparation of P3 GLAd (at least 3 × 10^9^ BFU) did not contain any adenovirus and/or RCA contaminants.

Additionally, we directed our attention toward P4 and P5 GLAd3.LacZ (1 × 10^8^ BFU for each) for the analysis of adenovirus and RCA. We prepared P4 and P5 GLAd utilizing P3 and P4 GLAd, respectively, as described (Fig. 3). We infected HEK293 cells with P4 or P5 GLAd pretreated with Benzonase, which removes the helper plasmid but not the capsid-protected adenovirus or RCA, and allowed the continued amplification of any potential adenovirus and RCA for additional rounds (P4: one round from P3; P5: two rounds from P3) (Fig. 4c). Similar to P3 GLAd, we were unable to detect any adenoviral species (either adenovirus or RCA) in the PCR-based analysis (Fig. 4d). Taken together, our data clearly demonstrate that our helper plasmid-based GLAd production system does not generate adenovirus and/or RCA as undesirable contaminants.

**Table 2:** Comparison of AAV and conventional GLAd with new GLAd

	Conventional GLAd	New GLAd
Use of helper adenovirus in production	Yes	No
Use of helper plasmid in production	n/a	Yes
Helper adenovirus contamination	Yes	No
RCA contamination	Yes	No
	**AAV**	**New GLAd**
Delivery capacity for transgene	~4.5 kb	~36 kb
Transduction efficiency^a^	+++ (70%)	+++++ (100%)
Broad tropism	Yes	Yes
Random integration into host genome (potential of insertional mutagenesis)	Yes^b^	No
Expression of viral proteins	No	No
In vivo acute toxicity by viral capsid	Yes	Yes
In vivo chronic toxicity	No	No
Long-term in vivo transgene expression	Yes	Yes

*n/a* not applicable

^a^Relative activity

^b^Although frequency is low

### The new GLAd as an efficient in vitro and in vivo gene delivery vector

Following all the successes described above, we attempted to test the gene delivery activity of our recombinant GLAd in vitro and in vivo. We chose *huntingtin* (9.4 kb) and *dystrophin* (11 kb) as target transgenes, as it is very difficult for other viral vectors to deliver these large genes and gene therapy has long been pursued as a treatment option for the associated diseases (HD and DMD). For proper packaging, GLAd genome size from the 5′ ITR to the 3′ ITR should be within the range of 27–37.8 kb, which is 75–105% of the original genome size^38,39^. Thus, we first converted the pGLAd3 genome plasmid to pGLAd4 (Fig. S8) to reduce its size because the length of pGLAd3 harboring large genes is near the upper length limit for virus packaging.

HD, which is inherited in a dominant fashion, is a fatal neurodegenerative disease caused by a poly-CAG (a codon for glutamine) repeat expansion in *huntingtin* gene^40^. HD patients possess one mutant copy of this gene, and the disease conditions can be ameliorated when the expression of mutant *huntingtin* is inhibited by an anti-sense oligonucleotide^41^ or RNAi^42,43^. Although the normal function of *huntingtin* in adult nerve cells remains unknown, it is important to note that *huntingtin* is essential for early embryonic development^44–46^ (*huntingtin* knockout causes embryonic lethality in mouse). Additionally, overexpression of wild-type huntingtin has been shown to reduce the cellular toxicity of mutant huntingtin^47^. These results suggest that a more appropriate recombinant GLAd for testing is to deliver the RNAi and *huntingtin* gene sequence together concurrently rather than delivering the *huntingtin* gene alone.

**Fig. 5:**
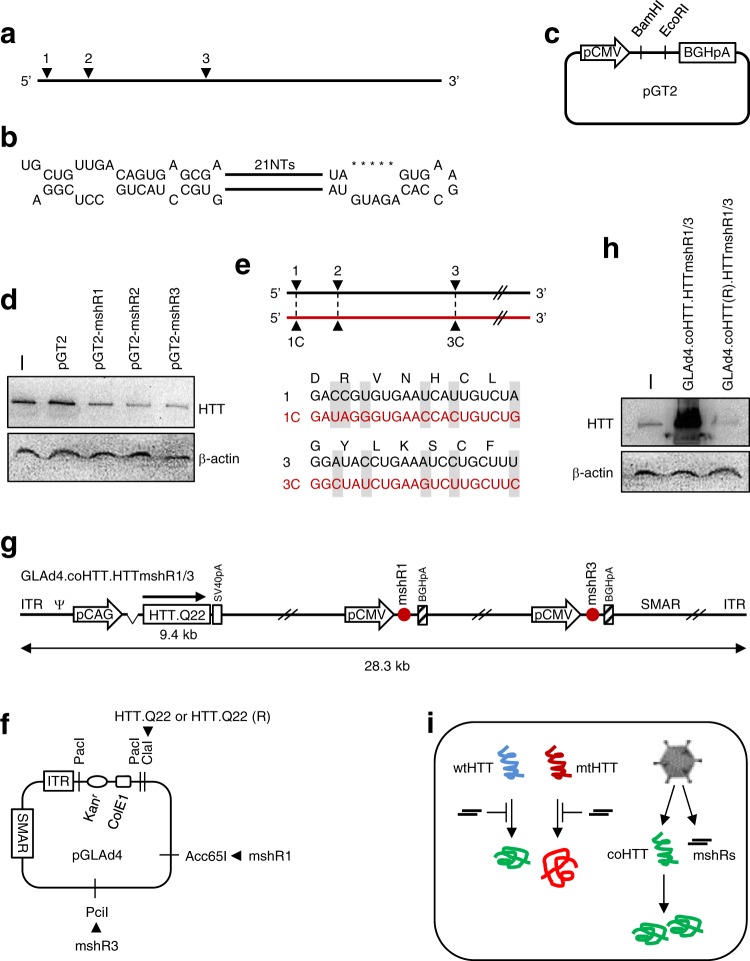
Expression of huntingtin mshRs, the codon-optimized synthetic *huntingtin* gene, or both by recombinant GLAd. **a** Schematic illustration of the full-length human mature *huntingtin* mRNA and the locations of mshRs. The numbers indicate the locations of the target sites of mshR1, mshR2, and mshR3. **b** Template for mshRs. The 21NTs are sense and anti-sense sequences of 21 nucleotides in length. **c**, Expression plasmid for mshRs. Individual mshR (Table S2) was cloned into this plasmid using the *Bam*HI and *Eco*RI sites. **d** Inhibition of endogenous huntingtin expression by mshR expression plasmids. HEK293T cells were either left untreated or were transfected with pGT2, pGT2-mshR1, pGT2-mshR2, or pGT2-mshR3. Forty-eight hours later, the cells were harvested and subjected to western blotting analysis of endogenous huntingtin expression. HTT denotes huntingtin. **e** Schematic illustration of the full-length human mature *huntingtin* mRNA and the locations of mshRs. The upper black and lower red horizontal lines indicate the native and codon-optimized *huntingtin* mRNAs, respectively. The numbers indicate the locations of the target sites of mshR1, mshR2, and mshR3. For mshR1 and mshR3, the amino acids and their corresponding codons are shown. Nucleotides identified as different between the native (endogenous) and codon-optimized huntingtin mRNA are highlighted with gray. **f** pGLAd4 genome plasmid for cloning mshR1, mshR3, and the codon-optimized synthetic *huntingtin* gene (right orientation or reverse (R) orientation). **g** Recombinant GLAd delivering both *huntingtin* mshRs and the codon-optimized synthetic *hungtingtin* gene simultaneously. GLAd4.coHTT.HTTmshR1/3 delivers the right-oriented codon-optimized hungtingtin gene. The total length (28.3 kb) is the size from the 5′ ITR to the 3′ ITR. **h** Effects of mshR1/3 and the codon-optimized synthetic *huntingtin* gene on huntingtin expression when simultaneously delivered by recombinant GLAd. At 48 h after the infection of HEK293T cells with GLAd4.coHTT.HTTmshR1/3 or GLAd4.coHTT(R).HTTmshR1/3 (reverse oriented *huntingtin* gene), the cells were harvested and subjected to western blotting for the analysis of huntingtin expression. HTT denotes huntingtin. **i** Action model of recombinant GLAd4.coHTT.HTTmshR1/3 in HD. wtHTT and mtHTT indicate wild type and mutant *huntingtin* mRNA, respectively. coHTT denotes mRNA transcribed from the codon-optimized synthetic *huntingtin* gene. Recombinant GLAd4.coHTT.HTTmshR1/3 simultaneously delivers both mshRs and the codon-optimized synthetic *huntingtin* gene to target HD tissues

For RNAi, we established miRNA-based shRNAs (mshRs herein) instead of conventional shRNAs to knock down the expression of *huntingtin* since mshRs have been proven to be safer than shRNAs for the knockdown of *huntingtin*^48^. We chose three target sites (Fig. 5a) that have been confirmed to be functional^48^ and constructed mshR expression constructs using synthetic mshRs (Fig. 5b, Table S2) and pGT2 plasmids (Fig. 5c). As expected, the transfection of HEK293T cells with each of these constructs resulted in decreased expression of endogenous huntingtin (Fig. 5d). Thus, we inserted two mshR expression cassettes for mshR1 and mshR3 into the pGLAd4 genome plasmid via PCR with appropriate primer sets (Table S3). For the *huntingtin* gene, we took into consideration that the poly-CAG repeats are the only difference between wild-type and mutant *huntingtin* genes. Thus, a knockdown approach can simultaneously reduce the expression of both endogenous wild type and mutant huntingtin proteins. To decrease concern regarding undesirable toxicity that might result from decreased expression of endogenous wild-type huntingtin, and also to compensate for the decrease in wild-type huntingtin, we utilized a codon-optimized synthetic *huntingtin* gene whose expression cannot be attenuated by mshRs (Fig. 5e). Through iHoA, we successfully transferred a codon-optimized synthetic gene expression cassette in different orientations into the pGLAd4 genome plasmid, in which both the mshR1 and mshR3 expression cassettes had already been inserted (Fig. 5f). We then produced recombinant GLAd4.coHTT.HTTmshR1/3 (Fig. 5g) and GLAd4.coHTT(R).HTTmshR1/3 viruses. The GLAd4.coHTT.HTTmshR1/3 virus overexpressed huntingtin, whereas the GLAd4.coHTT(R).HTTmshR1/3 virus inhibited the expression of endogenous huntingtin (Fig. 5h). Our data indicate that our recombinant GLAd successfully delivered both the mshRs and the codon-optimized synthetic *huntingtin* gene (~13 kb in total length) simultaneously (Fig. 5i). This recombinant GLAd can potentially be utilized as a gene therapy for the treatment of HD and is currently being investigated for clinical application.

In addition to recombinant GLAd delivering the large *huntingtin* gene, we have successfully constructed recombinant GLAds for many other small-sized transgenes, such as *factor IX* (R338L Padua mutant, for hemophilia B), *glucocerebrosidase* (GCR, for Gaucher’s disease), *hexosaminidase A* (HEXA, for Tay-Sachs disease), *hypoxanthine phosphoribosyltransferase* 1 (HPRT1, for Lesch-Nyhan syndrome), *iduronate-2-sulfatase* (IDS, for Hunter syndrome), *methyl-CpG-binding protein* 2 (MECP2, for Rett syndrome), and *survival of motor neuron* 1 (SMN1, for spinal muscular atrophy (SMA)). Each of these recombinant GLAds efficiently overexpressed the transgene in vitro (data not shown) and is also currently under investigation for possible clinical applications.

**Fig. 6:**
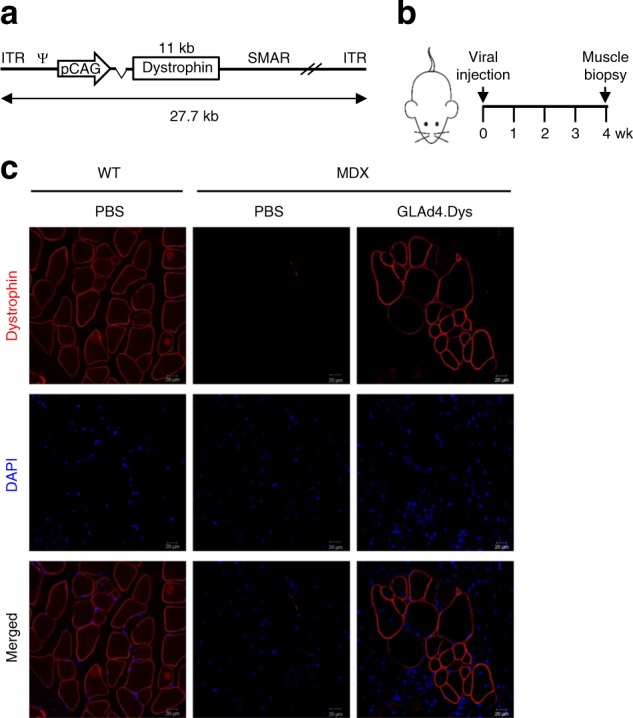
Recombinant GLAd delivers the *dystrophin* gene in vivo. **a** Schematic illustration of recombinant GLAd4.Dys. The total length (27.7 kb) is the size from the 5′ ITR to the 3′ ITR. **b** Time frame of animal experiments. **c** Examination of dystrophin expression in *dystrophin*-knockout MDX mice. The focal gastrocnemius muscle of MDX mice was injected with PBS or 50 μl of the GLAd4.Dys virus (4 × 10^10^ viral particles). Four weeks later, muscle tissues biopsied from the wild-type control and MDX mice treated with PBS or the GLAd4.Dys virus were subjected to immunofluorescence staining and analyzed under a confocal microscope (magnification = ×200; scale bar = 20 μm)

For in vivo study, we produced a recombinant GLAd that delivers the *dystrophin* gene (~11 kb), a target for DMD. We first cloned the human *dystrophin* gene into pBest4, constructed a recombinant pGLAd4_Dys plasmid through iHoA, and produced the recombinant GLAd4.Dys virus (Fig. 6a). We then injected the GLAd4.Dys virus into the focal gastrocnemius muscle of a *dystrophin*-knockout MDX mouse. Four weeks later (Fig. 6b), the muscle tissue at the injected site was biopsied and analyzed for dystrophin expression (Fig. 6c). Our recombinant GLAd4.Dys virus evidently expressed dystrophin in the target tissue. Although improvement of the disease condition was unobservable (unlike humans, MDX mice do not show manifestations of the disease), our data indicate that our GLAd4.Dys virus maintained the expression of dystrophin in the target tissue for at least 4 weeks.

## Discussion

An ideal in vivo gene delivery vector for gene therapy is recommended to exhibit the following characteristics: absence of random integration into the host genome (eliminating concern about insertional mutagenesis), no expression of viral proteins (requiring gutless viral genome, thus resulting in limited host immune response), broad tropism, high transduction efficiency in transgene delivery. and a sufficient cargo capacity for transgenes. Among the many gene delivery vectors currently available, GLAd is the only one that exhibits all of these recommended features^10–13^. Surprisingly, however, GLAd has only been used in animal studies so far, and no clinical applications have been attempted, thus, no clinical data for GLAd are currently available^14–20^. It is believed that this unexpected consequence is associated with the safety concerns raised by adenovirus, which is required for GLAd packaging and further amplification as a helper in the conventional GLAd production process, remaining as a contaminant in prepared GLAd. An additional hurdle to overcome for the clinical use of GLAd is the RCA contaminant^21^ generated during conventional GLAd production procedures or large-scale preparation of helper adenovirus.

The commonly used replication-incompetent first-generation adenovirus is highly immunogenic and can cause cellular toxicity in host organisms^20^. Thus, this virus has been widely used as a gene delivery vector for anti-cancer therapy, in which its immunogenic activity provides an adjuvant function and helps to remove tumor tissues more effectively through cooperation with the delivered therapeutic anti-cancer gene. In contrast, adenovirus is harmful in gene therapy, especially for the treatment of inherited genetic diseases requiring long-term transgene expression, since the host immune response caused by adenovirus can result in decreased or short-term transgene expression and toxicity in the patient. In this regard, it is important to note that the best result regarding adenovirus contamination^22^, even in the most advanced helper adenovirus-based GLAd production system, is 0.01%, which corresponds to 1 × 10^6^ out of 1 × 10^10^ viral particles.

Regarding safety measures, compared with the replication-incompetent first-generation adenovirus (the helper adenovirus used for GLAd production is usually a first-generation adenovirus), RCA is more dangerous. Unlike the first-generation adenovirus, which is devoid of E1, thus allowing replication only in E1-expressing packaging cells such as HEK293 and HEK293T cells, RCA can replicate autonomously in any cell types following its infection. In particular, when gene therapy contaminated with RCA is administered to an immunosuppressed (or immune-weakened) patient, RCA can easily replicate by itself, which could result in fatal toxicity in the patient. Furthermore, the E1 protein expressed from RCA can support the robust replication/amplification of the replication-incompetent adenovirus by complementing E1 deficiency of this adenovirus. Thus, the FDA heavily controls RCA and requires that RCA particles should be quantified in every batch of clinical adenovirus (and possibly also GLAd), and a single clinical dose should contain less than one infectious RCA particle in 3 × 10^10^ adenoviral particles. Therefore, it is clear that for the clinical use of GLAd, helper adenovirus should be replaced with a new helper and that a new GLAd production system completely free of concerns about adenovirus and RCA contaminants should be established, as demonstrated for the three plasmid-based AAV production system^49^, a standard procedure for preparing clinical AAV.

In this study, we report a novel helper plasmid for GLAd production for the first time. Our helper plasmid does not contain any ITRs and Ψ packaging signal, both of which are essential for viral packaging. Additionally, unlike helper adenovirus, our helper plasmid possesses only a single homologous region (nucleotides 3034–5015 (1482 bp) of adenovirus type 5, based on GenBank AC_000008) for homologous recombination, which can occur in HEK293 or HEK293T GLAd packaging cells. These structural characteristics not only eliminate the possibility of the conversion of the helper plasmid to active viral particles but also inhibit the generation of RCA during GLAd production. Actually, we were unable to detect any adenovirus or RCA contaminants even in the large-scale production of GLAd and in high-passage GLAd prepared through consecutive amplifications. Our data clearly indicate that our new helper plasmid-based GLAd system exclusively produces target recombinant GLAd completely free of adenovirus and RCA contaminants, which therefore significantly decreases the safety concerns raised by these contaminant viruses.

Although our helper plasmid cannot replicate in GLAd packaging cells, its helper function for GLAd packaging and further amplification is adequate to produce a sufficient amount of recombinant GLAd. In large-scale GLAd production, our system exhibited a moderately reduced efficiency compared with a conventional helper adenovirus-based system^22^. Nevertheless, our system allowed large-scale GLAd production with no difficulty. In drug development, safety is generally far more important than production yields. Such a consideration fully justifies the somewhat disadvantageous characteristics regarding the production yield of our GLAd system.

Our recombinant GLAd effectively delivered many transgenes in vitro and in vivo. As expected, our GLAd efficiently transferred large genes such as *huntingtin* (9.4 kb) and *dystrophin* (11 kb) or even multiple expression cassettes (~13 kb in total length) composed of the *huntingtin* gene and two mshRs. In many studies, the AAV-mediated delivery of the *dystrophin* gene has been explored, but with a focus only on the mini- or micro-*dystrophin* gene^50-53^, which are smaller versions of the full-length *dystrophin* gene exceeding the packaging capacity of AAV. It is important to note that our successful GLAd-mediated delivery of the multiple expression cassettes containing of *huntingtin* genes and two mshRs represents the first demonstration of a possible therapeutic approach for HD. These highlighted examples recapitulate a unique characteristic of GLAd regarding its versatility for gene delivery, particularly for the delivery of large genes.

The use of viral vectors in in vivo gene therapy is a double-edged sword. Viral vectors transfer transgenes more efficiently but can cause toxicity in host organisms. Gutless viral vectors such as AAV and GLAd do not contain any viral genes in their genome backbones; hence, they are safer than other viral vectors expressing viral proteins. Nevertheless, capsid protein-mediated toxicity of these vectors is unpreventable, as observed even for AAV, which exhibits severe acute toxicity at a high dose^54^. However, we can significantly decrease concern about virus capsid-mediated toxicity by utilizing a GLAd that is not contaminated with highly immunogenic adenoviral species or by using a significantly reduced amount of GLAd based on the safe delivery procedure demonstrated by balloon occlusion catheter-mediated intervention for GLAd-based liver-directed transgene expression^18,19^. This procedure exhibits a high safety profile and achieves long-term transgene expression for as long as 7 years^19^. In this context, our work needs to be evaluated, as we present a new platform for GLAd in regard to contamination of helper adenovirus and RCA for the first time and describe its outstanding characteristics as an in vivo gene delivery vector (Table 2).

In conclusion, our data clearly demonstrate that our own helper plasmid-based system efficiently produces recombinant GLAd that is free of adenovirus and RCA contaminants. Currently, gene therapy is addressing unmet needs for the treatment of various inherited genetic diseases. In this particular type of gene therapy, delivery vectors play a pivotal role, and we hope that our helper plasmid and GLAd production system will pave the way for the successful development of future GLAd-based gene therapy.

## Supplementary information

Supplementary Information
