# Supplementary material for: No more helper adenovirus: production of gutless adenovirus (GLAd) free of adenovirus and replication-competent adenovirus (RCA) contaminants
Source: Exp Mol Med. 2019 Oct 28;51(10):127. doi: 10.1038/s12276-019-0334-z (PMC6817846; doi:10.1038/s12276-019-0334-z)
Supplement: Supplementary file 1 — Supplementary Information [file 12276_2019_334_MOESM1_ESM.docx]

**Figure Legends for Supplementary Figures and Tables**

**Fig. S1. Construction of the pAdBest_dITR helper plasmid and the pBest cloning shuttle plasmid.** **a,** Schematic illustration of Ψ5, a derivative of Ad5. Vertical dotted lines in E1 and E3 indicate actual deletion locations in the corresponding gene. The unique BamHI is shown. **b-e,** Construction scheme for the pAdBest_dITR helper plasmid. For details, see Materials and Methods. **f,** Structural configuration of the cloning shuttle plasmid pBest. Black and gray boxes indicate the 5’ and 3’ homologous stretches for iHoA.


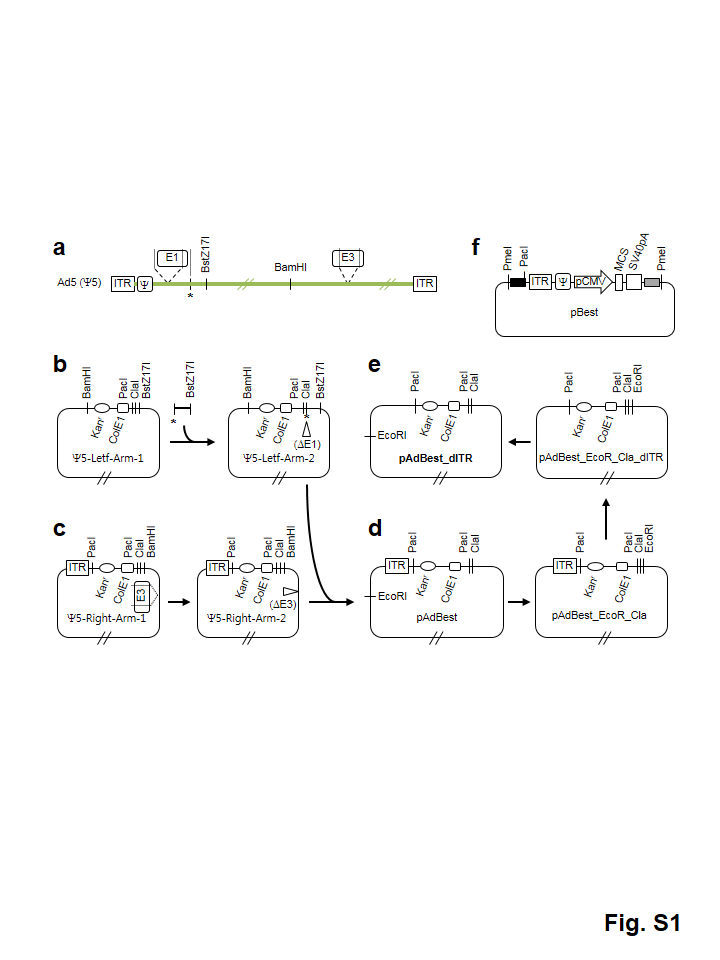


**Fig. S2. Construction of** **the pGLAd genome plasmid.** For details see Materials and Methods.

**
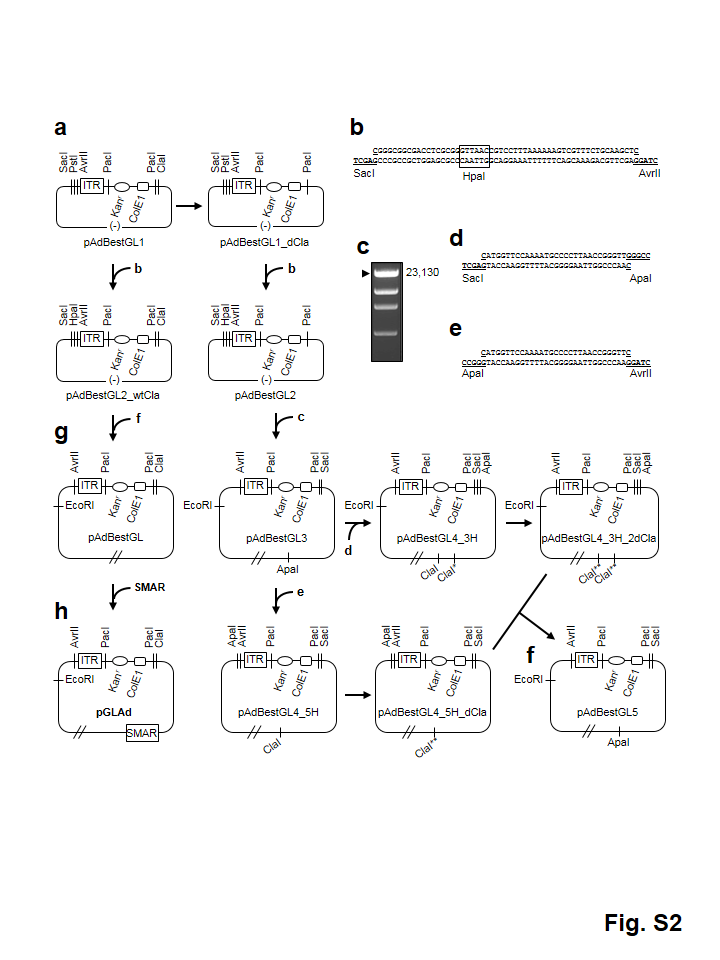
**

**Fig. S3. Schematic illustration of iHoA for the construction of recombinant pGLAd_LacZ.** **a,** The black boxes indicate PmeI sites, and arrowheads indicate the cleavage position of PmeI. PmeI-cleaved pBest_LacZ was mixed with ClaI-cut pGLAd and subjected to iHoA. 68 bp and 49 bp indicate the 5’ and 3’ homologous stretches, respectively, between pBest and pGLAd. **b,** Intermediate result of iHoA. Aligned double-stranded DNAs (a) were converted from single-stranded ones. This process resulted in hybrid-annealing and transferred the 5’ ITR, Ψ packaging signal and LacZ expression cassette to the pGLAd genome plasmid. **c,** Result of completed iHoA. The annealed strands are repaired in transformed bacterial cells.

**
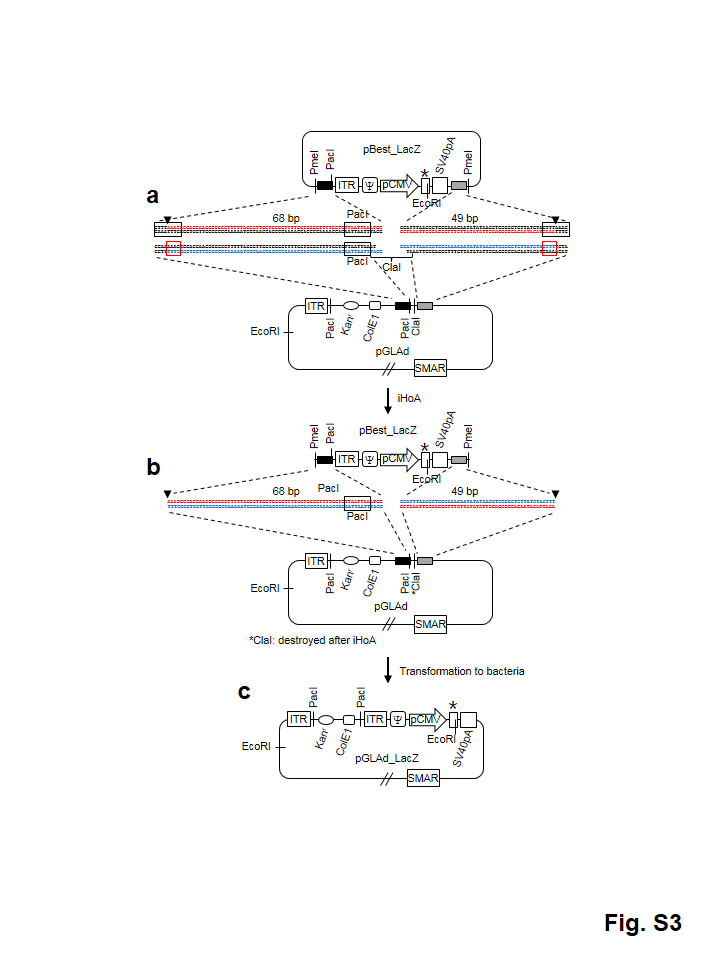
**

**Fig. S4. Efficiency of iHoA in the construction of recombinant pGLAd_X.** **a,** Bacterial colonies formed on an agar plate following iHoA. The arrows indicate smaller colonies, potentially containing the correct recombinant pGLAd_X plasmids. **b,** The restriction map for pGLAd_LacZ. E and B indicate EcoRI and BamHI sites, respectively. Numbers denote the locations of the corresponding restriction sites. **c,** Colony screening result. Five smaller colonies were picked and subjected to plasmid purification. The plasmids were digested with EcoRI and resolved on an agarose gel. M is the lambda HindIII size marker. **d,** Additional restriction digestion results. Clones 1 and 2 were doubly digested with EcoRI and BamHI. M is the lambda HindIII size marker.


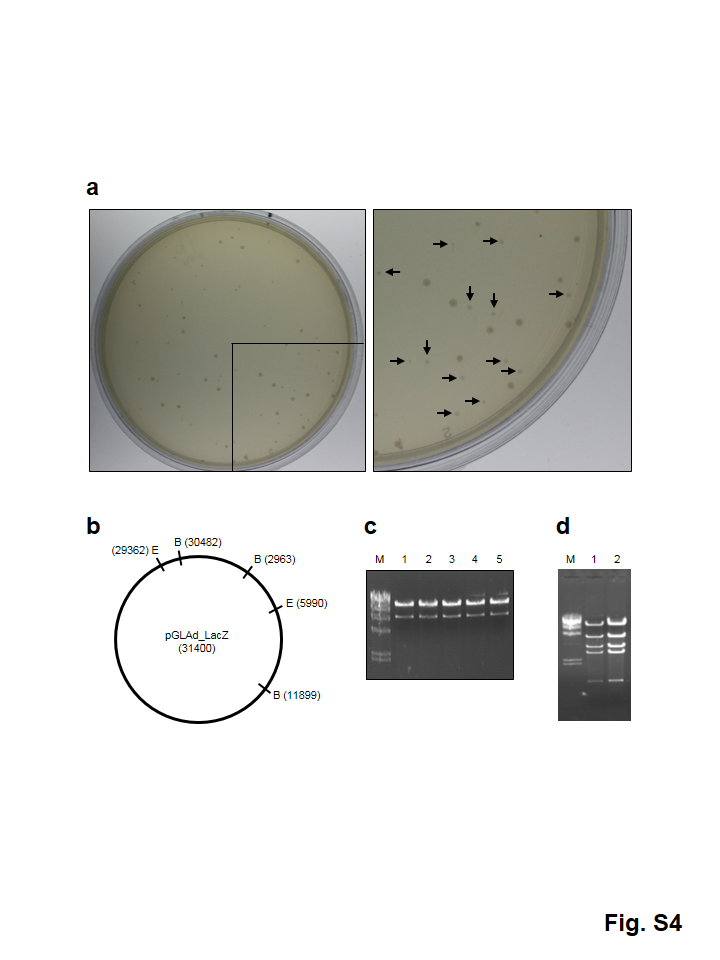


**Fig. S5. Sequence verification of iHoA junctions.** **a,** iHoA result. The colored arrowheads indicate sequencing primers. The arrowhead points to the starting nucleotide of the ClaI site (5’-ATCGAT-3’, destroyed after iHoA) used for the linearization of pGLAd in iHoA. The arrows indicate the ends of the homologous stretches used for iHoA. **b,c,** Sequencing results. The junction points are boxed. The arrows and arrowhead indicate the corresponding position described in (a).


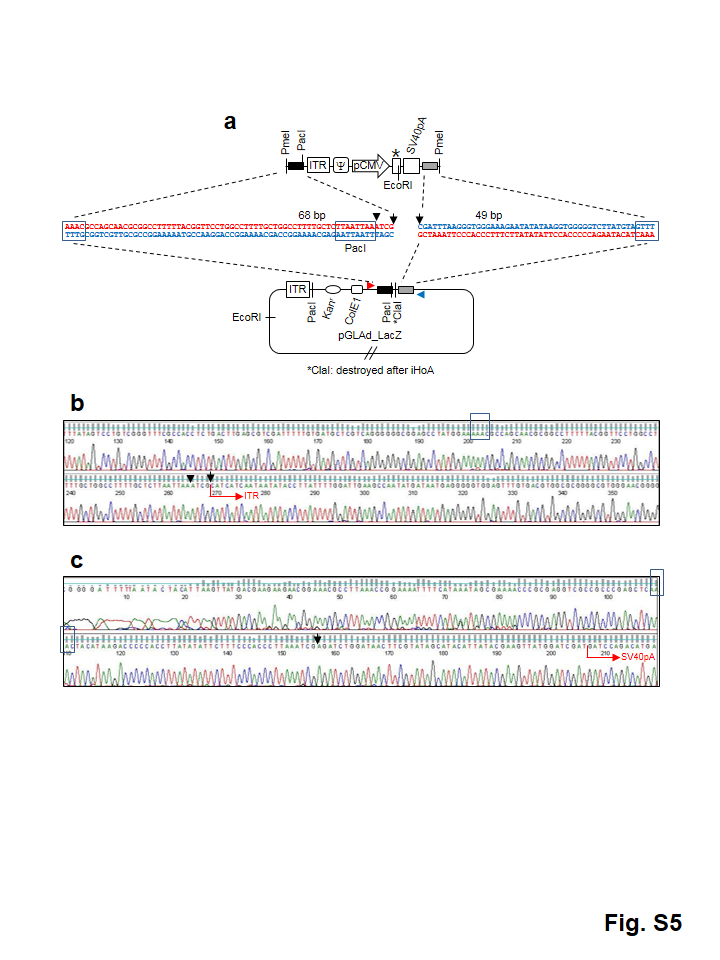


**Fig. S6.** **Construction of the pGLAd3, a new genome plasmid. a,** Schematic illustration of the mouse E-cadherin intron 2 region. The vertical black boxes indicate exons of the E-cadherin gene. The horizontal lines show the PCR products obtained using the primer sets (Table S1). N, C and F1-F5 represent the names of the PCR products. The numbers on the horizontal lines indicate the length of the PCR products. **b,** Construction scheme for the pGLAd3. For details, see Materials and Methods. **c,** Schematic illustration of the pBest4 cloning shuttle plasmid. Black and gray boxes indicate the 5’ and 3’ homologous stretches for iHoA. MCS represents the multi-cloning sites for transgenes.

**
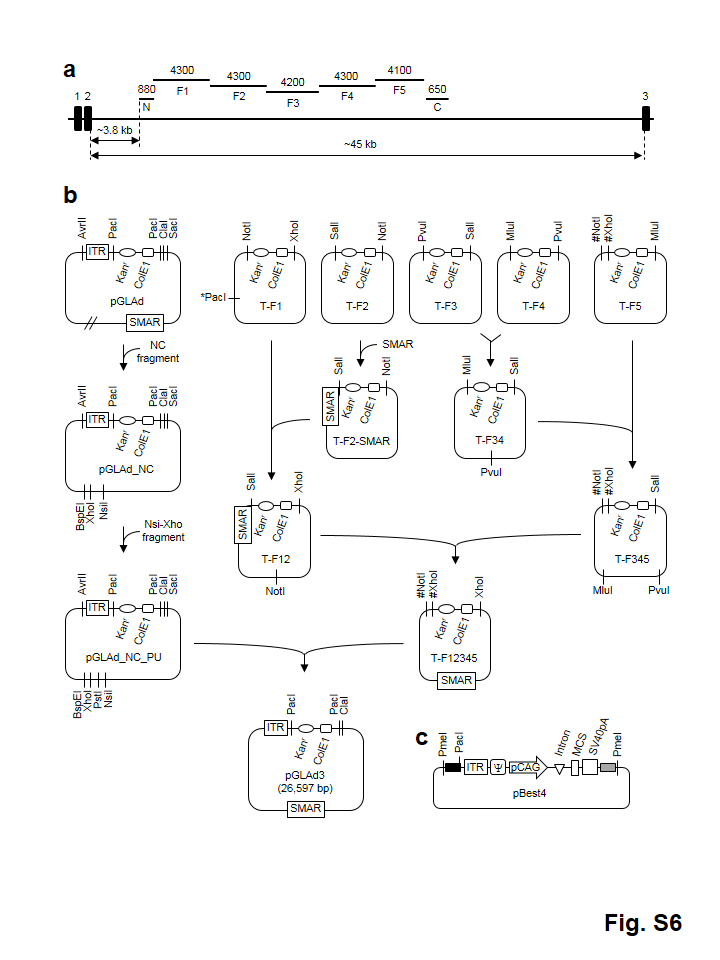
**

**Fig. S7. Two kinds of helpers for GLAd production and their effects on the generation of adenovirus and RCA contaminants.** Schematic representation of GLAd production with helpers in HEK293T or HEK293 packaging cells. Homologous recombination can occur in the colored boxed regions. RCA represents replication-competent adenovirus.

**
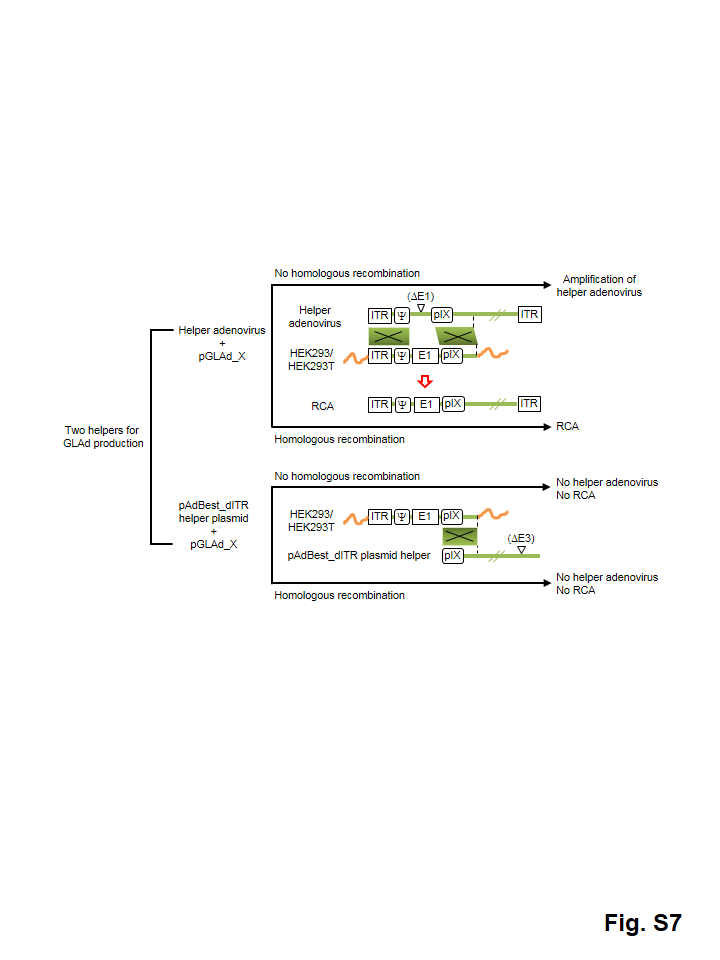
**

**Fig. S8. Conversion of the pGLAd3 into the pGLAd4 genome plasmid.** The pGLAd3 contains two BssHII sites. The pGLAd3 was digested with BssHII and self-ligated. This process deleted the portion indicated with the asterisk, decreasing the total length from 26,597 bp to 16,392 bp.


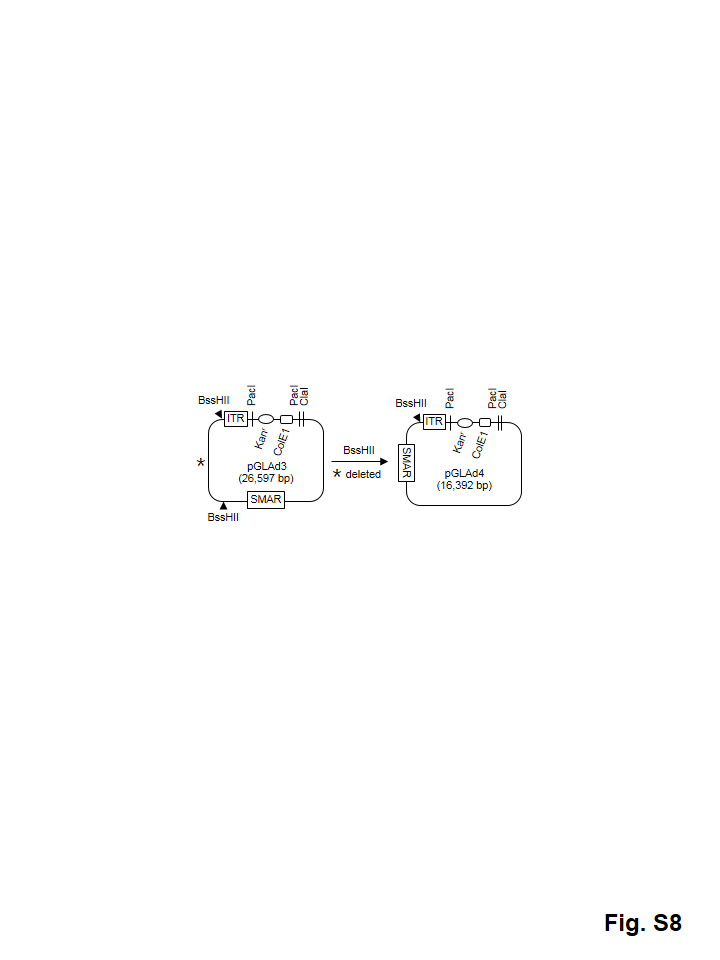


Table S1. Primers for cloning the pGLAd3 backbone

Name

Sequence

N-F

N-R

F1-F

F1-R

F2-F

F2-R

F3-F

F3-R

F4-F

F4-R

F5-F

F5-R

C-F

C-R

SMAR-F

SMAR-R

5’-TCTTATGTAGTTTGAGCTCGGCTTTGGTTATTTGATGGATTGACC-3’

5’-TCCGGAGGGGCTCGAGGCTGGCTTAAAACAGTAACTCAATATG-3’

5’-CTCGAGCCCCTCCGGAGGAGTGGCCAGGGCGTTCTGGAGGTAG-3’

5’-CGCCAAAAACCTAGGTGGGCCCAGGAAGACTCACTGGTTGG-3’

Nsi-Xho-S

Nsi-Xho-AS

5’-TCGAGCTAGACTCTGGGGCTAAAGCTGCAGTATCCATCACACTGGCGGCCGC-3’

5’-TCGAGCGGCCGCCAGTGTGATGGATACTGCAGCTTTAGCCCCAGAGTCTAGCTCGATGCA-3’

5’-CCTCCTCGAGCTAGACTCTGGGGCTAAAGCAATGAG-3’

5’-CCCACGCGTGCGGCCGCTTGAGAGGCAGAGGCAAAGGCAAGTG-3’

5’-TCAAGCGGCCGCGTGCTGAGACTAAAGGGATGTGCTAC-3’

5’-CCCGTCGACCATCATTCTAAGGCCTGCCTGAGCTA-3’

5’-CCCGTCGACGCACTGGCTTTACAGGGGCCGTCTGC-3’

5’-GGGCGATCGGGAAGGAAACCTACCTAGCCTACAAG-3’

5’-GGGCGATCGGTAGACTAGGATAGCCTCAAACTCCT-3’

5’-GGGACGCGTGTTAACATACTATCAGAATAGTGATA-3’

5’-GGGACGCGTTGGGTGCAATCTTACCAGAGCCTTAC-3’

5’-ACGGCAGTTCAAAATCAAGTAATAC-3’

5’-CGGCCTGGGTGGCCAAATAAACTTATAAATTGTGAGAGAAA-3’

5’-CAGGTCGACATATTTAAAGAAAAAAAAATTGTATC-3’

Table S2. Synthetic DNAs for expressing HTTmshRNAs

Name

Sequence

HTTmshR1_S

5’-**GATCC**TGCTGTTGACAGTGAGCGAGACCGTGTGAATCATTGTCTATAGTGA

AGCCACAGATGTATAGACAATGATTCACACGGTCGTGCCTACTGCCTCGGA**G**-3’

5’-**AATTC**TCCGAGGCAGTAGGCACGACCGTGTGAATCATTGTCTATACATCTG

TGGCTTCACTATAGACAATGATTCACACGGTCTCGCTCACTGTCAACAGCA**G**-3’

HTTmshR1_AS

5’-**GATCC**TGCTGTTGACAGTGAGCGACAGCTTGTCCAGGTTTATGAATAGTGA

AGCCACAGATGTATTCATAAACCTGGACAAGCTGGTGCCTACTGCCTCGGA**G**-3’

HTTmshR2_S

HTTmshR2_AS

5’-**AATTC**TCCGAGGCAGTAGGCACCAGCTTGTCCAGGTTTATGAATACATCTG

TGGCTTCACTATTCATAAACCTGGACAAGCTGTCGCTCACTGTCAACAGCA**G**-3’

5’-**GATCC**TGCTGTTGACAGTGAGCGAGGATACCTGAAATCCTGCTTTTAGTGA

AGCCACAGATGTAAAAGCAGGATTTCAGGTATCCGTGCCTACTGCCTCGGA**G**-3’

HTTmshR3_S

HTTmshR3_AS

5’- **AATTC**TCCGAGGCAGTAGGCACGGATACCTGAAATCCTGCTTTTACATCTG

TGGCTTCACTAAAAGCAGGATTTCAGGTATCCTCGCTCACTGTCAACAGCA**G**-3’

The bold nucleotides at 5’ and 3’ show cohesive ends for BamHI and EcoRI restriction sites, respectively.

Table S3. PCR primers for cloning HTTmshRs to pGLAd3 backbone

Name

Sequence

pGLAd3_5139Acc_CMV_AF_F

5’-ATTTTAAAGGGCCACGGTACGACATTGATTATTGACTAGTTATTAA-3’

pGLAd3_5139Acc_BGH_AF_R

5’-TGTGTCCATCCGTGTGGTACCCATAGAGCCCACCGCATCCCCAGCA-3’

pGLAd3_7189Pci_CMV_AF_F

5’-TTAACCCCACTCCCCACATGGACATTGATTATTGACTAGTTATTAA-3’

pGLAd3_7189Pci_BGH_AF_R

5’-GCATCTGAACGAAGCACATGCCATAGAGCCCACCGCATCCCCAGCA-3’

Underlined are nucleotide sequences homologous with pGLAd3 genome plasmid
